# Supplementary material for: Knowledge mobilisation: an ethnographic study of the influence of practitioner mindlines on atopic eczema self-management in primary care in the UK
Source: BMJ Open. 2019 Jul 26;9(7):e025220. doi: 10.1136/bmjopen-2018-025220 (PMC6661925; doi:10.1136/bmjopen-2018-025220)
Supplement: Author's manuscript [file bmjopen-2018-025220.draft_revisions.pdf]

BMJ Open is committed to open peer review. As part of this commitment we make the peer review history of every article we publish publicly available.

When an article is published we post the peer reviewers' comments and the authors' responses online. We also post the versions of the paper that were used during peer review. These are the versions that the peer review comments apply to.

The versions of the paper that follow are the versions that were submitted during the peer review process. They are not the versions of record or the final published versions. They should not be cited or distributed as the published version of this manuscript.

BMJ Open is an open access journal and the full, final, typeset and author-corrected version of record of the manuscript is available on our site with no access controls, subscription charges or pay-per-view fees (http://bmjopen.bmj.com).

If you have any questions on BMJ Open's open peer review process please email info.bmjopen@bmj.com

BMJ Open

Knowledge mobilisation: An ethnographic study of the influence of practitioner mindlines on eczema self-management in primary care in the United Kingdom

Journal:	*BMJ Open*
Manuscript ID	bmjopen-2018-025220
Article Type:	Research
Date Submitted by the Author:	04-Jul-2018
Complete List of Authors:	Cowdell, Fiona; Birmingham City University, Faculty of Health Education and Life Sciences
Keywords:	Eczema < DERMATOLOGY, Ethnography, Knowledge mobilisation, Mindlines, PRIMARY CARE, Self-management

SCHOLARONE™
Manuscripts

For peer review only

BMJ Open KM Practitioner mindlines 4.7.18 for submission

Article title: Knowledge mobilisation: An ethnographic study of the influence of practitioner mindlines on eczema self-management in primary care in the United Kingdom

Author information:
Fiona Cowdell
DProf, RN
Professor of Nursing and Health Research
Faculty of Health, Education and Life Sciences
Birmingham City University
220 Ravensbury House
Westbourne Road
Edgbaston
Birmingham
B15 3TN
UK
Telephone: +44 (0)121 300 4345
Email: Fiona.cowdell@bcu.ac.uk
orcid.org/0000-0002-9355-8059

Word count: 4802

Number of figures and tables: one figure and two tables

Keywords: eczema, ethnography, knowledge mobilisation, mindlines, primary care, self-management

Disclaimer and source of support:
This report is independent research arising from a Knowledge Mobilisation Research Fellowship, Professor Fiona Cowdell, KMRF-2015-04-004 supported by the National Institute for Health Research. The views expressed in this publication are those of the authors and not necessarily those of the NHS, the National Institute for Health Research, Health Education England or the Department of Health.

For peer review only

BMJ Open KM Practitioner mindlines 4.7.18 for submission

Knowledge mobilisation: an ethnographic study of the influence of practitioner mindlines on eczema self-management in primary care in the United Kingdom

Abstract

Objective: To explore how eczema specific mindlines are developed by primary care practitioners.

Design: Ethnographic study.

Setting: One large, urban general practice in central England.

Participants: In observation, all practitioners and support staff in the practice and in interviews a diverse group of practitioners (n=16).

Results: Observation of over 250 hours and interview data were combined and analysed using an ethnographic approach through the lenses of mindlines and self-management. Three themes were identified: beliefs about eczema, eczema knowledge and approaches to self-management. Eczema mindlines are set against a backdrop of it being a low priority and not managed as a long-term condition. Practitioners believed that eczema is a simple to manage with little change in treatments available and prescribing limited by local formularies. Practice is largely based on tacit knowledge and experience. Self-management is expected but not often explicitly facilitated. Clinical decisions are made from knowledge accumulated over time. Societal and technological developments have altered the way in which practitioner mindlines are developed; in eczema, for most, they are relatively static.

Conclusions: The outstanding challenge is to find novel, profession and context-specific, simple, pragmatic strategies to revise or modify practitioner mindlines by adding reliable and useful knowledge and by erasing outdated or inaccurate information thus potentially improve quality of eczema care.

Strengths and limitations

- First ethnographic study to examine the development of eczema specific mindlines
- Diverse sample primary care practitioners
- Ethnographer was a lone researcher
- Results may be context specific

For peer review only - http://bmjopen.bmj.com/site/about/guidelines.xhtml

Introduction

Eczema is a common, long-term skin condition affecting around one in five children and one in twelve adults in the United Kingdom (UK). It can have a detrimental impact on wellbeing and quality of life and globally is one of the fifty most burdensome diseases (1). Eczema is mainly treated in primary care (2). People may seek advice from general practitioners (GPs), practice nurses (PNs), nurse practitioners (NPs), health visitors (HVs), community pharmacists (CP) and pharmacy counter staff (PCS).

GP consultations are often unsatisfactory for both patient (3) and practitioner (4, 5) with GPs dominating encounters and using avoidance tactics (6) and there being significant dissonance between patient / parent and GP beliefs about assessment and treatment (6). Many GPs have limited specialist dermatology knowledge (7). Nurse consultations, albeit in secondary care, tend to be more positively evaluated (8, 9) and minimal research has been conducted into the contribution of HVs. Research into the role of the CP in dermatology care is limited (10) and expertise may be suboptimal (11) despite CPs reporting being at least reasonably confident in their role (12). The role of the pharmacy counter assistant is equally under-researched although they are often first point of contact for customers and may offer health advice independent of pharmacists (10, 13).

The mainstay of eczema treatment is the regular application of emollients, at least daily and often for many years, with or without intermittent topical steroids and calcineurin inhibitors. Non-adherence results from the high self-management demand of applying topical treatments (14) but also lack of information and conflicting advice from different health professionals (15). Despite available evidence (for example the National Institute for Health Research Guideline for Eczema (16) and the Global Resource for Eczema Trials database 17)), providing evidence-based treatment appears to be a challenge for health professionals managing eczema (18).

Self-management is a policy imperative which can improve disease outcomes and quality of life for people living with long-term conditions (19). Strategies to support eczema self-management are poorly understood,

For peer review only - http://bmjopen.bmj.com/site/about/guidelines.xhtml

have limited availability, can be costly and have variable impact (20). Eczema is not classified as a long-term condition in the same way as other illnesses for example asthma (21).

Primary care practitioners are expected to deliver evidence-based practice (EBP). Evidence based medicine (EBM) was originally the preserve of doctors and was defined as “the conscientious, explicit, and judicious use of current best evidence in making decisions about the care of individual patients” (22). Over time, other professions have embraced EBP but this has, at times, been conceptualised as a set of research-based facts which if disseminated to practitioners will ensure more standardised, high quality care (23); this notion is now largely dismissed (24). Primary care practitioners face particular challenges in EBP given the volume of information they need and information overload is a real problem (25).

The study of knowledge mobilisation (KM) is growing exponentially in health care, at its simplest it is “moving knowledge to where it can be most useful” (26). KM involves determined efforts to create, share and use research and other forms of knowledge predicated on the understanding that to be effective KM activity must be relational, constructed from social interaction and context-specific (27-29).

Mindlines, developed from a primary care based ethnographic study (30) offer a “real world” approach to mobilising knowledge and changing clinical practice. Mindlines are “collectively reinforced, internalised tacit guidelines” which underpin clinical decision-making (30). They build on the work of Polyani (31) and Nonaka and Takeuchi (32) who propose that knowledge is not necessarily conscious and explicit, and that tacit knowledge in the form of unconscious schemata and technical know-how are dominant influencers of action compared with formal codified knowledge. Gabbay and LeMay (28) suggest that mindlines are based on flexible, embodied and intersubjective understanding of knowledge that is grounded in the acceptance that there are multiple realties and that knowledge is context-specific. Mindlines represent a complex amalgamation of knowledge gathered from many sources for example, communication with colleagues and opinion leaders in the field and from tacit knowledge developed over time (28). In their original work, Gabbay and LeMay (30) examined the construction of mindlines across primary care. A subsequent

For peer review only - http://bmjopen.bmj.com/site/about/guidelines.xhtml

synthesis of 10 years of mindline literature (n=340) reports that they have been conceptualised and used in four distinct ways. "Nominal" in which the term was used in name only, sometimes with a degree of scepticism, "in practice" examining how mindlines are developed and spread in everyday practice, "theoretical and philosophical" in which the aim was to extend existing theory and "solution focused", exploring ways in which mindlines can be influenced. Solution focused papers (n=28) emphasise the importance of collaborative learning, relationship building and effective leadership in the development of valid, collective, evidence-based mindlines. This review reveals a paucity of information about development or strategies to amend condition specific mindlines (33). Repeating the search strategy utilised for this review in 2018 revealed an abundance of further related literature but little directly addressing condition specific mindlines or how they may best be amended.

Given the prevalence of eczema, the challenges of primary care consultations and the high self-management demand, it is prudent to investigate the way in which eczema mindlines are constructed by practitioners. This will inform understanding of mindlines "in practice" and will underpin future "solution focused" work to develop novel, context-specific, simple and pragmatic strategies to revise or modify eczema mindlines by adding reliable and useful knowledge and by erasing outdated or inaccurate information thus potentially improve quality of eczema care and self-management.

Method

Aim

To understand construction of healthcare practitioner eczema mindlines in primary care.

Design

An ethnographic approach was employed. Ethnography is founded in anthropology and is concerned with the systematic study of people and cultures (34). Data is collected through extensive observation with informal conversations, field notes and interviews (35, 36). Data was collected in one large general practice in England.

For peer review only - http://bmjopen.bmj.com/site/about/guidelines.xhtml

Setting, participants and process

Data were collected by the author, a nurse and researcher, from January - June 2017. The General Practice was identified by a local clinical research network. It was a research and education active urban general practice in a demographically diverse and deprived area of England with a patient population of approximately 10,000. Observations were also conducted in a local pharmacy. No practitioners reported a special interest in dermatology. In preparation for data collection the researcher attended two practice meetings to outline conduct of the study. Data were collected in more than 250 hours of observation during all surgery opening hours. The role of social-participant-as-observer, that is, predominantly observer with some social functions such as cleaning couches was taken (37). Observation began with the reception team to understand the day-to-day working of the practice. Observation of consultations with GPs, GP trainees and locums, nurses, health visitors in baby clinics and pharmacy staff followed. GP telephone consultations were listened to and discussed with the practitioner. Field notes were documented and informal conversations either written contemporaneously or audio-recorded. Entire clinics were attended regardless of presenting complaint, to gain understanding in the context of other long-term conditions. Between consultations practitioners recounted recent eczema consultations. Single, semi-structured interviews using a topic guide (Table 1) were conducted with practitioners from each profession (n=16) (Table 2) using maximum variation purposive sampling (38) to ensure a mix of job role and level of experience. A predominance of female participants was reflective of the profile of the healthcare team.

Table 1: Practitioner interview topic guide

- Do you have any special interest in skin health?
- How much contact do you have with patients with eczema?
- What sort of treatments do you use most often?
- How do you decide on a particular treatment?
  - What impact does the local formulary have on your prescribing?
- How much are you able to advise patients on how to care for their eczema?
  - Concordance etc

For peer review only - http://bmjopen.bmj.com/site/about/guidelines.xhtml

- How do you update your own knowledge about eczema?
- How could we best get research information to use in your practice?
  - What methods do you use now?
  - Can you give any specific examples?
- Do patients come with their own ideas about the treatment they need?
- How much do you and your patient share the decision about what treatment to use?
- How do you reconcile patient's needs with what is available?
- Do you refer patients to any external sources of information?

Table 2: Demographic details of interview participants

Role	Years in practice
Health visitor	10
GP	35
GP Trainee	2
Practice nurse	31
Practice nurse	32
Pharmacist	8
GP Trainee	5
Pharmacist	12
Pharmacy counter staff	10
Pharmacy counter staff	17
GP Trainee	7
GP	6
GP	5
Health visitor	2
Health visitor	2

For peer review only - http://bmjopen.bmj.com/site/about/guidelines.xhtml

Health visitor	3

Interviews were conducted in the workplace and lasted from 22-40 minutes. Data sufficiency was achieved when no new insights were forthcoming (39). For completeness documents and websites were reviewed including the NICE Clinical Guideline for Eczema (17), the local emollient formulary and the Clinical Knowledge Summary (40) and GP notebook pages (41) for eczema.

Data collection and analysis were iterative with initial findings being used to guide further collection (42). Audio-data were professionally transcribed and transcripts read against the recording by the researcher to confirm accuracy. Data analysis was completed independently by the researcher, though the lenses of mindlines and self-management. Transcripts and field notes were read in full to get a sense of the data as a whole, and then manually coded, categorised and merged into themes. Post theme development, relevant sections of the data were revisited to ensure authentic interpretation and use of participant language.

Reflexivity

Reflexivity was maintained throughout the study with particular attention being paid to subjectivity and positioning as a nurse and skin health researcher; pre-understandings were consciously set aside (43).

Patient and Public Involvement

Lay people were involved in the development of the research question and in planning the design of the study.

Results

Data analysis resulted in three themes: beliefs about eczema; eczema knowledge and approaches to self-management. Each is discussed with examples from the data below.

Theme 1: Beliefs about eczema

For peer review only - http://bmjopen.bmj.com/site/about/guidelines.xhtml

Eczema was consistently viewed as a "*bread and butter*" [GP] condition that accounted for many consultations. However, although 19.5% of the practice population was recorded as having some type of eczema few consultations primarily for this condition were observed. Analysis of patient reported reason for GP consultation for a typical week during observation revealed that 26/627 (4.1%) of reasons were skin related with none citing eczema as the primary complaint. No observed face-to-face consultations were primarily for eczema; it was reported as a secondary concern in a small number of number of GP consultations and more often to HVs in baby clinics. This resulted in eczema necessarily being given limited attention "*it's often a secondary problem and there's only time to deal with one problem per consultation"* [GP]. Telephone consultations with GPs were witnessed and patients were observed to consult with pharmacy staff about their eczema. Practitioners mainly viewed eczema as a nuisance condition requiring limited knowledge to treat effectively, "*eczema is simple to treat, nothing much has changed over the years"* [GP] and "*the recipe doesn't change* [GP]".

Some GPs described eczema as a "*catch up"* [GP] consultation when clinics were over-running. GPs and nurses noted the absence of specific external incentives for long-term eczema management and that it was a condition without the "*red flags"* [GP] which trigger treatment escalation or referral. They described treatment options as straightforward involving emollients with or without intermittent topical steroids. Few mentioned calcineurin inhibitors or other available medications. Most practitioners considered emollients to be a homogenous group of preparations all with similar properties, although a few differentiated in terms of viscosity and texture. Pharmacy staff and HVs were familiar with a broader range of emollient products and were more likely to offer suggestions for over the counter preparations. This was in part because no HVs in this study were able to prescribe. GPs were reluctant to prescribe topical steroids or other treatments unless absolutely necessary. PNs rarely saw patients with eczema other than older people with varicose eczema who were attending for leg ulcer treatment.

Practitioners recognised that eczema could have a negative impact on wellbeing and quality of life but this was not often reflected in the care offered. Treatment was mainly in reaction to a flare rather than there

For peer review only - http://bmjopen.bmj.com/site/about/guidelines.xhtml

being a long-term plan of care. Generally patients were able to access regular repeat prescriptions for emollients and practitioners expressed a level of frustration when they presented with a flare having not requested or used the prescribed treatments. Although 'safety netting' was always in place, planned follow-up consultations were not suggested. Empathy for patients was most evident in practitioners who had personal experience of eczema, they articulated a varying level of understanding about the differences between products, regardless of available empirical evidence, and the extent to which personal preference influenced concordance. Pharmacy counter staff were the most conversant with the differences between emollient products having tried samples, and they were most likely to share this knowledge with patients / customers verbally and in leaflets.

Although eczema was viewed as a frequent reason for consultation, it was mainly presented as a secondary concern and so dealt with swiftly. Eczema was considered simple to treat with little change over time although practitioners with personal experience of eczema were more aware of the challenges of self-management and tolerant of personal treatment preferences.

Theme 2: Eczema knowledge

Beliefs about eczema influenced the formation of mindlines and for most mindlines were set against a backdrop of eczema being a low priority condition and a perception of unchanging treatment options which were constrained by local prescribing guidelines (Figure 1). Many practitioners described eczema as a common conditions for which you "*know [treatment] by heart* [GP]" and likened his response to using a "*satnav …………you stop thinking, the little NHS boxes [on the computer] tell you what to prescribe* [GP]".

Most practitioners reported that their eczema knowledge was based on their initial education and recognised "*pre-reg derm education was very, very basic"* [GP]. A few had completed dermatology placements during GP training but reported seeing little eczema. One experienced GP recounted learning from a consultant, her practice was unchanged as she had "*learnt from a consultant many years ago and never heard anything to contradict it"* [GP]. PNs and GPs were aware of available dermatology education

For peer review only - http://bmjopen.bmj.com/site/about/guidelines.xhtml

but did not attend as it was a low priority and costly, "*there is training but you have to pay*" [PN] and they preferred to "*avoid reps and sponsored sessions*" [GP]. HVs reported that skin health was never an educational priority. Pharmacist's knowledge was updated through e-bulletins from different sources and covered only changes in, and availability of, medications. Only PCS received eczema specific education by attending regular seasonal sessions provided by their employer. Although deemed to be useful, particularly as they tried products and were advised on correct application, the educational experience was sometimes suboptimal as one reported how she was "*shamed into remembering*" [PCS] session content.

Local emollient guidelines underpinned many prescribing decisions so practitioners did not need to think as "*software will fire up a message if another product should be used*" [CP]. Changes to guidelines were ascribed to cost and "*what was in vogue*" [GP]. Practitioners were not concerned about these changes stating for example, "*aqueous cream, they've gone off that idea for some reason*" [PCS] and "*Zero products are the ones that are currently on trend*" [GP trainee]. Whilst some prescribers stuck rigidly to prescribing the cheapest product, "*I try to be good and prescribe the cheaper side of things*" [GP trainee], others were more flexible according to their own or the patient's preference. However, deviations from the formulary were rare on the basis that "*local formulary is very constraining and you'd have to be able to justify why you'd prescribed anything else*" [GP]. Exceptions were observed in the baby clinic and in pharmacy practice where patients were often informed about a wider range of emollients that could be purchased over the counter. For those who paid a prescription charge this could often be more cost effective. PCS suggested that they were able to advise patients readily as they had "*tried samples so you can tell the customers what they feel like*" [PCS].

Other knowledge sources contributed to eczema mindlines. All staff, with the exception of experienced GPs, used internet searches most commonly the online resources GP Notebook and Clinical Knowledge Summaries. Useful websites were often book-marked and visited in preparation for a consultation rather than alongside the patient. If information was not located almost immediately the practitioner switched to another website "*we're hard wired for speed now*" [GP] and "*dipped into what's relevant*" [GP] as and when

For peer review only - http://bmjopen.bmj.com/site/about/guidelines.xhtml

required. None mentioned existing NICE Eczema Guidelines. Local emollient guidelines existed and influenced the prescribing practice of most practitioners, however others were unaware of these and some found them hard to access. GPs and HVs used different emollient guidelines and this caused confusion for patients when they consulted both. A member of pharmacy staff noted the need for "*a synchronised approach so patients don't get confused"* [PCS]. Practitioners also experienced confusion when offering advice on treatment application, for example "*treatment is a bit arbitrary – for example should you advise steroid or emollient first?"* [HV].

Practitioners learnt from each other to a limited extent, most often within their professional groups. They recognised "*we learn both good and bad habits from each other"* [PN]. Opportunities for shared learning had reduced as there was little time to meet up and in-house teaching for GPs and PNs had "*fallen by the wayside"* [GP] due to staff sickness and pressure of work. One GP reported "*phoning a friend*", now a consultant dermatologist, when she needed advice. GPs reported learning from trainees during debrief sessions but could not recall ever having discussed eczema. Trainees exhausted all available information sources before seeking advice from a GP. HVs and PNs met more frequently and exchanged knowledge more regularly, although eczema was not a condition of interest.

Practitioners expressed varied views on the value of patient knowledge and experience and the extent to which it influenced care. PNs, HVs and pharmacy staff respectively reported that they routinely "*ask patient what they have tried already"* [PN], "*see what's worked for them"* [HV] and "*listen and learn from customers"* [PCS] and used this information as a basis for treatment advice. Others listened to patients with a degree of scepticism but acquiesced to patient preference, "*patients often have fixed ideas [about emollients] and I try to accommodate these"* [GP]. A few were less receptive, for example "*I try to use guidelines and the formulary ………… patient experience stuff can be counterproductive"* [GP trainee] and others suggested that their wider experience overrode the patients personal preferences and experiences "*experience wise I've found a lot of people get on with it [particular emollient]"* [GP] and therefore that was what would be prescribed.

For peer review only - http://bmjopen.bmj.com/site/about/guidelines.xhtml

Only the most experienced practitioners spontaneously articulated the existence of tacit knowledge stating, "*it's a perpetual exercise … adding on knowledge and skills"* [GP] and "*built up knowledge over time"* [PN]. Others pointed to more concrete sources of knowledge. All practitioners understood reliability of evidence to a greater or lesser extent.

Eczema knowledge was constructed from different sources by individual professions. Nursing and medical staff perceived a limited need to update their knowledge as eczema care was viewed as having changed little over time. Exceptions to this were practitioners who had personal experience of eczema and pharmacy staff who regularly updated their mindlines using informal and formal sources of knowledge.

Theme 3: Approaches to self-management

In principle, all practitioners supported self-management of eczema but recognised the difficulties of achieving this in practice particularly without formal recognition as a long-term condition (LTC). Some practitioners routinely used techniques to support self-management for patients with other LTCs. Strategies included for example by "*finding out patients' expectations"* [PN], "*tailoring knowledge to the person"* [GP], "*start with what the patient understands and then fill in the gaps"* [GP], "*give patients a map of management"* [GP], "*instil confidence"* [GP] and "*reinforce that self-management is good"* [GP trainee]. A few GPs used specific techniques such as "*short bursts of CBT"* [GP], "*motivational interviewing techniques ……. compressed to fit in consultation"* [GP] and "*behaviour modification ………… not a one consultation job"* [GP]. Even practitioners who did not articulate using strategies to support self-management integrated them in practice for many LTCs. However they were rarely observed or discussed in relation to eczema.

Most eczema care was reactive when patients presented with a flare and talk of eczema care was almost exclusively about treatment options with virtually no attention paid to ensuring that the patient understood the condition and actions they could take to avoid the relentless cycle of flares. The most tangible contribution to self-management was the availability of repeat prescriptions for emollients but advice to use these consistently was lacking. Barriers to self-management were observed, for example the

For peer review only - http://bmjopen.bmj.com/site/about/guidelines.xhtml

appointment system often precluded patients seeing the same GP over time so treatment could be altered without the benefit of fully understanding the patient journey to date. Contradictory advice given by practitioners and a lack of faith in patient's ability to judge when they needed to use topical steroids and to use them safely presented significant barriers to successful self-management. Practitioners suggested the "*need to see patients before prescribing [topical] steroids"* [GP], one GP stated that "*sensible*" patients may get steroids on repeat but struggled to quantify sensible in this context. Pharmacy staff did not recognise their contribution to self-management per se but recognised the positive impact they had on eczema management through *"actually taking notice of what they're telling me"* [PCS] and perceived "*they do trust me ………………… I'm well known in the local community"* [PCS] and were therefore easy for customers to speak with.

Whilst recognising the need for self-management the fact that eczema is not categorised as a long-term condition limited how much patients were supported to self-manage and at times healthcare systems could hinder attempts.

Discussion

This study offers new insights into how primary care practitioners construct eczema specific mindlines. Practitioner mindlines are predominantly set against a back drop of eczema being a low priority, due to a combination of not being viewed as an LTC and so lacking external incentives and the perception of available treatments being standard use of emollients and topical steroids, which changes little over time and is constrained by prescribing guidance. This led to an assumption that there was little need to amend mindlines. Eczema mindlines were developed early in their career by many practitioners and were relatively static amongst GPs, PNs and HVs, except for those with direct personal experience of eczema. Mindlines of pharmacy staff were regularly modified through a combination of education provided by their employer, electronic updates from professional bodies and interactions with customers. The latter was particularly influential for the PCSs as they generally had more time to listen and had built up trusting relationship with the customers over time.

For peer review only - http://bmjopen.bmj.com/site/about/guidelines.xhtml

This study is one of few to apply mindline theory to a specific condition across a broad range of practitioners. In particular it identifies important differences in the way in which eczema mindlines are developed and so may best be amended for individual practitioner groups. This study conforms with conventions of robust qualitative work in that it is rigorous (coherent and sufficiently well reported to be open to external audit), relevant (enriches understanding of the subject), resonant (resonates with readers experiences and understandings) and reflexive (subjectivity of the author is acknowledged) (44). Limitations include issues of reliability as the ethnographer is a lone worker, however this is mitigated by conversations with participants to check understandings. As data was collected in one general practice, findings may not be transferable but the diversity of participants should minimise this risk (45). Additionally no nurse practitioners were included as, at the time of data collection, none were employed in the practice.

As with the original conceptualisation of Gabbay and leMay (30), practitioner eczema mindlines are composed over time, from a range of evidence sources which rarely embrace direct use of research. Gabbay and le May (28) point to the critical nature of knowledge-in-practice-in-context in which in each context new knowledge is converted by the complex social processes of the Socialization, Externalization, Combination, Internalization spiral (32). Context was central in the formation of eczema mindlines but was informed more by long-held beliefs and national policy than by local context. Key differences in this study are that mindline development has evolved alongside the changing nature of primary care where practitioners, particularly GPs, appear to work more in isolation than as part of a community with "coffee room chat" (46) appearing much reduced. In parallel available online resources have spiralled thus potentially reducing the need to confer with others. This challenges the notion that mindlines are heavily reliant on professional interactions (28). The static nature of eczema mindlines and the beliefs underpinning eczema care meant that they were accessed using fast, automatic, System 1 thinking rather than the more deliberative, conscious slow and effortful System 2 approach (47).

For peer review only - http://bmjopen.bmj.com/site/about/guidelines.xhtml

Few studies have investigated condition specific mindlines with the exception of a Tanzanian study of malaria diagnosis (48), however the depiction here is more akin to rules of thumb or heuristics. A comprehensive commentary on mindlines identifies 76 papers categorised as "in practice", that is studies of how mindlines are developed, many of these used the term to mean consulting with colleagues (33). A smaller number were faithful to the original Gabbay and Le May's conceptualisation but add little by way of new understanding. More recently Wieringa and colleagues (49) investigated mindlines development in online clinical communities concluding that they offered collective, dynamic settings and implicitly that they may be areas for mindline amendment. Whilst online communities may appeal to some practitioners, this will not be so for all.

In this study eczema was consider low priority. These beliefs are longstanding with surveys suggesting that both patients and practitioners perceive dermatology as a poor relation in healthcare (50-52) and Magin and colleagues (4) describing 'dismissive' and 'unsympathetic' attitudes amongst GPs. Eczema appears to be considered as "health problem which is not an illness" (53) and therefore less legitimate and worthy than other conditions. Ambivalence about eczema specific learning was in contrast to a survey which indicated a desire for new knowledge, particularly in the form of education delivered by consultants (54); inevitably GPs completing the survey would be those with an interest in dermatology. The dermatology community has used many strategies to make research findings accessible to all with limited success (55). In contrast with this study in which treatment for eczema was viewed as simple others report GPs uncertainty about managing eczema (56).

Achieving change in primary care practice is challenging, interventions most likely to influence practice demonstrate evidence of benefit, are simple to use and adaptable to local context (57). The context of eczema mindlines, that it is a low priority condition with a limited repertoire of treatment options, is unlikely to change in the foreseeable future. If, like other LTCs eczema was recognised in the Quality and Outcomes Framework (58) patients may benefit from the accelerated trends towards systematic management (59). Practitioners in primary care are expert generalists (60) and are expected to have

knowledge of many conditions for which there is wealth of available evidence. This may lead to information overload for which coping strategies are needed. Bate and colleagues (61) describe "satisficing" that is, curtailing the amount of information gathered to enable them to make a "good enough" decision.

In many ways it can be argued that treatment of eczema in primary care is relatively straightforward and that amendment of mindlines to adjust thinking about emollients and removal of outdated information about topical steroid use could make a significant change in practice that would improve both patient experience and self-management practices. Brevity and accessibility of information is key as practitioners have been found to judge the usefulness of new knowledge as function of its relevance x validity ÷ by the work needed to access it (62). It is possible that straightforward messages could be conveyed through media such as aphorisms, "succinct sayings that offer advice" (63) or actionable nuggets "knowledge translation tools designed to provide …….. concise practical information about the most prevalent and pressing primary care needs of patients" (64). This approach offers the opportunity to compensate for the loss of professional wisdom through personal communication by transmitting concentrated wisdom and guidance in a different way (63).

Efforts to amend GPs, PNs and HVs mindlines need to be accessible via rapid System 1 thinking. Interventions should be specific, practical, tailored, relevant and rapidly delivered information which can readily be assimilated, or as participants in this study described it, a "no faff" approach. Given their time constraints and information gathering habits any new information would best be delivered individually rather than in a group setting and available online and possibly in other formats.

The role of the community pharmacist in eczema care is evolving partly in response to Pharmaceutical Services Negotiating Committee guidance on Medicines Use Reviews (65), New Medicine Service (66) and Minor Ailment Service (67). Forthcoming changes in availability of emollients on prescription may increase their role further. Pharmacy staff described eczema mindline development as a more collective experience than other practitioners and valued learning from each other and from customers. They may be open to

For peer review only - http://bmjopen.bmj.com/site/about/guidelines.xhtml

group approaches to update and remove redundant information from their mindlines and this would need to be brokered through both professional and employing organisations.

Conclusion

This ethnographic study provides new understandings about the development of eczema specific mindlines in different practitioner groups in primary care. The outstanding challenge is to find novel, context-specific, simple, pragmatic strategies to revise or modify these mindlines by adding reliable and useful knowledge and by erasing outdated or inaccurate information using strategies that are most appropriate to each profession. Mindline amendment has the potential to improve self-management and quality of eczema care through the delivery of consistent, evidence-based care.

For peer review only

For peer review only - http://bmjopen.bmj.com/site/about/guidelines.xhtml

BMJ Open KM Practitioner mindlines 4.7.18 for submission

Contribution statement

FC is the sole contributor to this paper

Funding

This report is independent research arising from a Knowledge Mobilisation Research Fellowship, Professor Fiona Cowdell, KMRF-2015-04-004 supported by the National Institute for Health Research. The views expressed in this publication are those of the authors and not necessarily those of the NHS, the National Institute for Health Research, Health Education England or the Department of Health.

Ethical approval

The study was approved by a National Health Service REC (16/YH/0252). Process consent was used for observation, on each occasion informal conversations were used to re-check participant's willingness to be observed. Patients were informed about the study by practitioners and when necessary the researcher exited individual consultations, either at the request of the patient, the practitioner or using personal judgment, although this was infrequently needed. Written consent was taken for audio-recorded interviews. Interview participants consented to publication of anonymised information.

Competing interests

None to declare

Acknowledgements

Thanks to Amanda Roberts who have given invaluable lay feedback on the planning and design of this study. Thanks to James Mycock, Birmingham City University for the mindline illustration and to Professors Hywel William and Stephen Timmons, University of Nottingham for their valuable feedback on earlier iterations of this manuscript

Data sharing statement

The datasets generated and/or analysed during the current study are not publicly available as they are not designed to be re-analysed by others but are available from the corresponding author on reasonable request.

For peer review only

References

1. Hay RJ, Johns NE, Williams HC, et al. The global burden of skin disease in 2010: an analysis of the prevalence and impact of skin conditions. J Invest Dermatol. 2014; 134: 1527-1534.
2. Schofield J, Grindlay D, Williams H, Skin conditions in the UK: a health care needs assessment. Centre of Evidence Based Dermatology, University of Nottingham; 2009.
3. Cowdell F. Knowledge mobilisation: an ethnographic study of the influence of lay mindlines on eczema self-management in primary care in the United Kingdom. BMJ Open. In press
4. Magin P, Adams J, Heading G, Pond C. Patients with skin disease and their relationships with their doctors: a qualitative study of patients with acne, psoriasis and eczema. Med J Aust. 2009; 190:62-64
5. Santer M, Burgess H, Yardley L, et al. Experiences of carers managing childhood eczema and their views on its treatment: A qualitative study. Br J Gen Pract. 2012; 62:261-267.
6. Powell K, Le Roux E, Banks J, et al. GP and parent dissonance about the assessment and treatment of childhood eczema in primary care: a qualitative study. BMJ Open. 2018; 8:e019633.
7. Rübsam M, Esch M, Baum E, et al. Diagnosing skin disease in primary care: A qualitative study of GPs' approaches. Fam Pract. 2015; 32: 591-595.
8. Nicol NH, Ersser SJ. The role of the nurse educator in managing atopic dermatitis. Immunol Allergy Clin. 2010; 30:369-383.
9. Rolinck-Werninghaus C, Trentmann M, Reich A, et al. Improved management of childhood atopic dermatitis after individually tailored nurse consultations: a pilot study. Pediat Allergy Immunol. 2015; 26: 805-810.
10. Tucker R, Stewart D. Why people seek advice from community pharmacies about skin problems. Int J Pharm Pract. 2015; 23(2): 150-153.
11. Tucker RP, MacLure K, Paudyal V, et al. An exploratory study of community pharmacist diagnosis and management of dermatitis and acne. Selfcare J. 2017; 8(2):1-10
12. Tucker R. The medicines use review in patients with chronic skin diseases: are pharmacists doing them and how confident are they? Int J Pharm Pract. 2013; 21:202-204.
13. Banks J, Shaw A, Weiss MC. The community pharmacy and discursive complexity: a qualitative study of interaction between counter assistants and customers. Health Soc Care Community. 2007; 15:313-321.
14. Santer M, Burgess H, Yardley L, et al. Managing childhood eczema: Qualitative study exploring carers' experiences of barriers and facilitators to treatment adherence. J Adv Nurs. *2013*; 69:2493-2501.
15. Teasdale EJ, Muller I, Santer M. 2017. Carers' views of topical corticosteroid use in childhood eczema: a qualitative study of online discussion forums. Br J Dermatol. 2017; 176(6):1500-1507.
16. Global Resource for Eczema Trials (GREAT) database http://www.greatdatabase.org.uk/GD4/Home/Index.php Accessed 4.7.18
17. National Institute of Health and Care Excellence. Atopic eczema in under 12s: diagnosis and management. Clinical guideline [CG57] 2007 https://www.nice.org.uk/Guidance/CG57. Accessed 4.7.18
18. Powell K, Le Roux E, Banks JP, et al. Developing a written action plan for children with eczema: A qualitative study. Brit J Gen Pract. 2018; 68(667):e81-e89.
19. Department of Health 2014 The Mandate: A mandate from the Government to NHS England: April 2014 – March 2015 https://www.gov.uk/government/uploads/system/uploads/attachment_data/file/383495/2902896_DoH_Mandate_Accessible_v0.2.pdf Accessed 4.7.18
20. Ridd MJ, King AJL, Le Roux E, et al. Systematic review of self-management interventions for people with eczema. Br J Dermatol. 2017; 177(3):719-734.
21. Hanlon P, Daines L, Campbell C. et al. Telehealth interventions to support self-management of long-term conditions: a systematic metareview of diabetes, heart failure, asthma, chronic obstructive pulmonary disease, and cancer. JMIR. 2017; 19:e172.

For peer review only - http://bmjopen.bmj.com/site/about/guidelines.xhtml

22. Sackett, DL, Rosenberg WM, Gray JM, et al. Evidence based medicine: what it is and what it isn't. BMJ. 1996; 312:71-2.
23. Greenhalgh T, Howick J, Maskrey N. "Evidence based medicine: a movement in crisis?" BMJ 348 (2014): g3725.
24. Brownson RC, Colditz GA, Proctor EK. eds. Dissemination and implementation research in health: translating science to practice. Oxford: Oxford University Press; 2017.
25. Vaucher C, Bovet E, Bengough T, et al. Meeting physicians' needs: a bottom-up approach for improving the implementation of medical knowledge into practice. Health Res Policy Syst. 2016; 14:49.
26. Ward V. 2017. Why, whose, what and how? A framework for knowledge mobilisers. Evid Policy. 2017; 13:477-497.
27. Ferlie E, Crilly T, Jashapara A, et al. Knowledge mobilisation in healthcare: a critical review of health sector and generic management literature. Soc Sci Med. 2012;74:1297-1304.
28. Gabbay J, Le May A. Practice-based evidence for healthcare: clinical mindlines. London: Routledge; 2010.
29. Plamondon K, Caxaj S. 2018. Toward Relational Practices for Enabling Knowledge-to-action in Health Systems: The Example of Deliberative Dialogue. Adv Nurs Sci. 2018; 41:18-29.
30. Gabbay J, le May A. Evidence based guidelines or collectively constructed "mindlines?" Ethnographic study of knowledge management in primary care. BMJ. 2004; 329:1013.
31. Polanyi M. The logic of tacit inference. Philos.1966; 41:1-18.
32. Nonaka I, Takeuchi H. The knowledge-creating company: How Japanese companies create the dynamics of innovation. Oxford: Oxford University Press; 1995.
33. Wieringa S, Greenhalgh T. 10 years of mindlines: a systematic review and commentary. Imp Sci. 2015; 10:45.
34. Hammersley M, Atkinson P. Ethnography: Principles in Practice. Abingdon: Routledge;2007.
35. Spradley JP. Participant observation. Illinois: Waveland Press; 2016.
36. Spradley JP. The ethnographic interview. Illinois: Waveland Press; 2016.
37. Cowdell F. That's how we do it we treat them all the same: An exploration of the experiences of patients, lay carers and health and social care staff of the care of people with dementia in acute hospital settings. Cambridge. Cambridge Publishing. 2013.
38. Tongco MD. Purposive sampling as a tool for informant selection. Ethnobotany Research and Applications. 2007. 31; 5:147-58.
39. O'Reilly M, Parker N. 'Unsatisfactory Saturation': a critical exploration of the notion of saturated sample sizes in qualitative research. Qual Res. 2013; 13:190-197.
40. Clinical Knowledge Summaries. Eczema – atopic https://cks.nice.org.uk/eczema-atopic Accessed 4.7.18
41. GP Notebook. Eczema http://www.gpnotebook.co.uk/simplepage.cfm?ID=-623902719 Accessed 4.7.18
42. Polkinghorne DE. Language and meaning: Data collection in qualitative research. J Couns Psychol, 2005; *52*(2):137.
43. Matua GA, Van Der Wal DM. Differentiating between descriptive and interpretive phenomenological research approaches. Nur Res. 2015; 22(6):22.
44. Finlay L. Phenomenology for psychotherapists: Researching the lived world. Oxford: Wiley Blackwell; 2011.
45. University of Hertfordshire 2017 Disadvantages of Ethnographic Research http://www.health.herts.ac.uk/immunology/Web%20programme%20-%20Researchhealthprofessionals/disadvantages_associated_with_et.htm. Accessed 4.7.18
46. Gabbay J, le May. Mindlines: making sense of evidence in practice. B J Gen Pract. 2016: 402-403.
47. Kahneman D. Thinking, Fast and Slow. London: Penguin; 2011.
48. Chandler CI, Jones C, Boniface G et al. Guidelines and mindlines: why do clinical staff over-dignose malaria in Tanzania? A qualitative study. Malar J 2008;7:53
49. Wieringa S, Engebretsen E, Heggen K, et al. How knowledge is constructed and exchanged in virtual communities of physicians: qualitative study of mindlines online. JMIR. 2018;20: e34.

For peer review only - http://bmjopen.bmj.com/site/about/guidelines.xhtml

50. Rosoff SM, Leone MC. (1989). The prestige of dermatologists. are they "last among equals"?Int J Dermatol. 1989; 28:377-380.
51. Rosoff SM, Leone MC (1991). The public prestige of medical specialties: Overviews and undercurrents. Soc Sci Med. 1991;32:321-326.
52. Creed PA, Searle J, Rogers ME. Medical specialty prestige and lifestyle preferences for medical students. Soc Sci Med. 2010;71:1084-8.
53. Cornwell J. Hard-earned lives: accounts of health and illness from East London:Routledge Kegan & Paul;1984.
54. Soriano LF, Sahota A, Jolliffe V. An evaluation of general practitioners learning preferences in dermatology. Education for Primary Care. 2017; 17:1-2.
55. Baron SE, Cohen SN, Archer CB. Guidance on the diagnosis and clinical management of atopic eczema. Clin Exp Dermatol. 2012; 37:Suppl 1, 7-12.
56. Le Roux, E, Powell K, Banks JP, et al. GPs' experiences of diagnosing and managing childhood eczema: a qualitative study in primary care. Br J Gen Pract. 2018. Feb 16:bjgp18X694529.
57. Lau R, Stevenson F, Ong BN, et al. Achieving change in primary care—causes of the evidence to practice gap: systematic reviews of reviews. Imp Sci. 2015;11:40.
58. National Institute for Health and Care Excellence (2018) NICE Quality and Outcomes Framework indicator https://www.nice.org.uk/standards-and-indicators/qofindicators Accessed 4.7.18
59. Roland M, Guthrie B. Quality and Outcomes Framework: what have we learnt? BMJ 2016;354:i4060
60. Reeve J, Dowrick CF, Freeman GK, et al. Examining the practice of generalist expertise: a qualitative study identifying constraints and solutions. JRSM short reports. 2013; 4(12):2042533313510155.
61. Bate L, Hutchinson A, Underhill J, et al. How clinical decisions are made. Br J Clin Pharmacol. 2012; 74:614-20.
62. Cunningham M, Shirley A. Mindlines in a digital age. Education for Primary Care. 2015; 26:293-6.
63. Levine D, Bleakley A. Maximising medicine through aphorisms. Med Educ. 2012; 46:153-162
64. McColl MA, Aiken A, Smith K, et al. Actionable nuggets: knowledge translation tool for the needs of patients with spinal cord injury. Can Fam Physician. 2015; 61:e240-e248.
65. Pharmaceutical Services Negotiating Committee Medicines Use Review http://psnc.org.uk/services-commissioning/advanced-services/murs/ Accessed 4.7.18
66. Pharmaceutical Services Negotiating Committee New Medicine Service http://psnc.org.uk/services-commissioning/advanced-services/nms/ Accessed 4.7.18
67. Pharmaceutical Services Negotiating Committee Minor Ailment Service http://psnc.org.uk/services-commissioning/locally-commissioned-services/en8-minor-ailments-service/ Accessed 4.7.18

BMJ Open KM Practitioner mindlines 4.7.18 for submission

w only

For peer review only - http://bmjopen.bmj.com/site/about/guidelines.xhtml

BMJ Open KM Practitioner mindlines 4.7.18 for submission

Table 1: Practitioner interview topic guide

Table 2: Demographic details of participants

Figure 1: Practitioner eczema mindlines

Sources of information underpinning practitioner eczema mindlines.

For peer review only

For peer review only - http://bmjopen.bmj.com/site/about/guidelines.xhtml

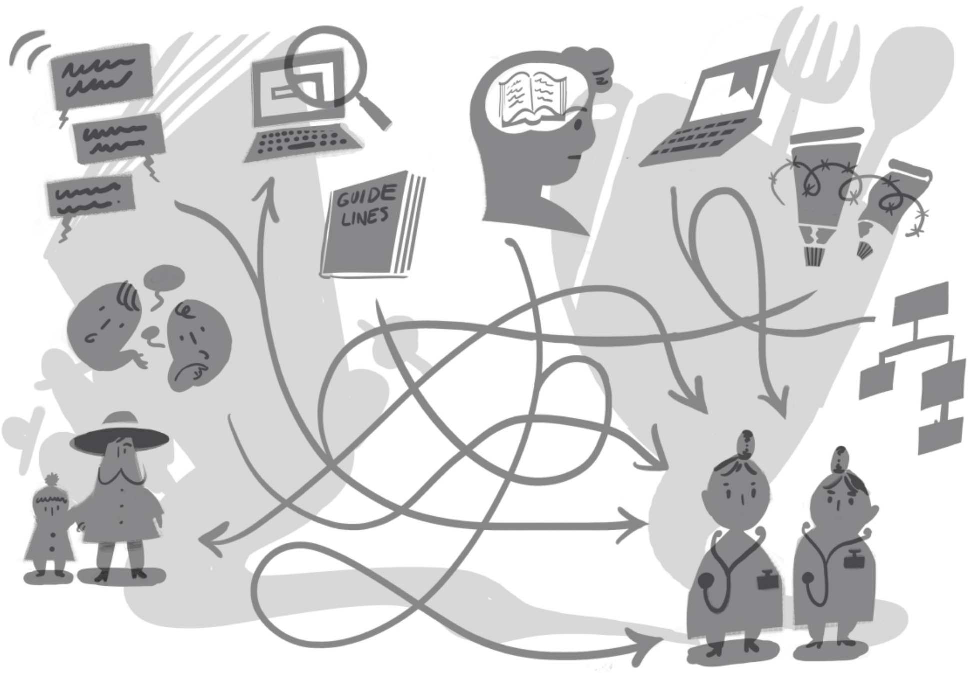

Practitioner eczema mindlines

742x524mm (120 x 120 DPI)

BMJ Open Practitioner eczema mindlines COREQ 4.7.18

COREQ Statement

Knowledge mobilisation: An ethnographic study of the influence of practitioner mindlines on eczema self-management in primary care in the United Kingdom

**Statement**	**Page no**
**Domain 1: Research team and reflexivity**<br>*Personal Characteristics*<br>1. Interviewer/facilitator Which author/s conducted the interview or focus group?<br>I conducted all observation and interviews	6
2. Credentials What were the researcher's credentials? E.g. PhD, MD<br>DProf, RN	Title page
3. Occupation What was their occupation at the time of the study?<br>Professor of Nursing and Health Research	Title page
4. Gender Was the researcher male or female?<br>Female	Title page
5. Experience and training What experience or training did the researcher have?<br>I have extensive experience in qualitative research.	Title page
Relationship with participants<br>6. Relationship established Was a relationship established prior to study commencement?<br>I established rapport with each observational participant at each meeting and with interviewees at the time of interview.	6
7. Participant knowledge of the interviewer<br>What did the participants know about the researcher? e.g. personal goals, reasons for doing the research<br>I met the team prior to observation, provided an information sheet, explained about myself and the study and answered questions. I gave interview participants an information sheet prior to interviews and answered any questions, I reiterated this information at the beginning of each interview.	6
8. Interviewer characteristics What characteristics were reported about the interviewer/facilitator? e.g. Bias, assumptions, reasons and interests in the research topic<br>Participants were aware, and it is reported in the manuscript, that I am a Registered Nurse with an interest in how eczema knowledge is developed and shared between patients and practitioners in primary care and that this was a publically funded study.	8
**Domain 2: study design**<br>*Theoretical framework*<br>9. Methodological orientation and theory<br>What methodological orientation was stated to underpin the study? e.g. grounded theory, discourse analysis, ethnography, phenomenology, content analysis<br>In the methods section I explain that this is an ethnographic study using observation and interviews and that data analysis followed an ethnographic approach through the lenses of mindlines and self-management.	5
Participant selection<br>10. Sampling How were participants selected? e.g. purposive, convenience, consecutive, snowball<br>I collected observational data during 250 hours in the practice. I selected interview participants using maximum variation purposive sampling to ensure a mix of profession, gender and years in practice.	6
11. Method of approach How were participants approached? e.g. face-to-face, telephone, mail, email<br>Observation participants were recruited at the beginning of each encounter with the practice. I recruited interview participants from the practice and local pharmacy.	6
12. Sample size How many participants were in the study?<br>Observation involved many participants and 16 interviews were completed	6
13. Non-participation How many people refused to participate or dropped out? Reasons?<br>On occasion I exited consultations at the request of the patient, practitioner or of my own volition<br>A few practitioners declined to take part in interviews due to time constraints.	5
Setting<br>14. Setting of data collection Where was the data collected? e.g. home, clinic, workplace<br>Observational data was collected in a GP practice. Interviews were conducted in their workplace.	6
15. Presence of non-participants Was anyone else present besides the participants and researchers?	6

For peer review only

BMJ Open Practitioner eczema mindlines COREQ 4.7.18

Observational data involved numerous individuals. Interviews were conducted individually.	
16. Description of sample What are the important characteristics of the sample? e.g. demographic data, date<br>Interview participants were sampled by profession, gender and years in practice.	Table 2
Data collection<br>17. Interview guide Were questions, prompts, guides provided by the authors? Was it pilot tested?<br>A topic guide was used for interviews	Table 1
18. Repeat interviews Were repeat interviews carried out? If yes, how many?<br>No repeat interviews were carried out	NA
19. Audio/visual recording Did the research use audio or visual recording to collect the data?<br>Interviews were audio recorded and observational data recorded in field notes	6
20. Field notes Were field notes made during and/or after the interview or focus group?	6
21. Duration What was the duration of the interviews or focus group?<br>Interviews lasted from 22-40 minutes	6
22. Data saturation Was data saturation discussed?<br>Data sufficiency was achieved when no new sources of knowledge were identified in interviews.	8
23. Transcripts returned Were transcripts returned to participants for comment and/or correction?<br>No	NA
**Domain 3: analysis and findings**<br>*Data analysis*<br>24. Number of data coders How many data coders coded the data?<br>I analysed the data independently and subsequently discussed with research, clinical and lay colleagues who corroborated initial interpretations.	8
25. Description of the coding tree Did authors provide a description of the coding tree?<br>No	NA
26. Derivation of themes Were themes identified in advance or derived from the data?<br>Themes were derived inductively from the data	8
27. Software What software, if applicable, was used to manage the data?<br>No	NA
28. Participant checking Did participants provide feedback on the findings?<br>No	NA
Reporting<br>29. Quotations presented Were participant quotations presented to illustrate the themes / findings? Was each quotation identified? e.g. participant number<br>Quotations are provided and profession of participant is identified.	9 - 14
30. Data and findings consistent Was there consistency between the data presented and the findings?<br>Yes.	9 - 14
31. Clarity of major themes Were major themes clearly presented in the findings?<br>Yes.	9 - 14
32. Clarity of minor themes Is there a description of diverse cases or discussion of minor themes?<br>Yes, I present a spectrum of practitioners and variations of mindlines.	9 - 14

For peer review only

BMJ Open

Knowledge mobilisation: An ethnographic study of the influence of practitioner mindlines on atopic eczema self-management in primary care in the United Kingdom

Journal:	*BMJ Open*
Manuscript ID	bmjopen-2018-025220.R1
Article Type:	Research
Date Submitted by the Author:	15-Mar-2019
Complete List of Authors:	Cowdell, Fiona; Birmingham City University, Faculty of Health Education and Life Sciences
**Primary Subject Heading**:	Dermatology
Secondary Subject Heading:	Evidence based practice, General practice / Family practice, Health services research, Qualitative research
Keywords:	Ethnography, Knowledge mobilisation, Mindlines, PRIMARY CARE, Self-management, Atopic eczema

SCHOLARONE™
Manuscripts

Clean copy BMJ Open KM Practitioner mindlines R1 15.3.19 2

Article title: Knowledge mobilisation: An ethnographic study of the influence of practitioner mindlines on atopic eczema self-management in primary care in the United Kingdom

Author information:
Fiona Cowdell
DProf, RN
Professor of Nursing and Health Research
Faculty of Health, Education and Life Sciences
Birmingham City University
220 Ravensbury House
Westbourne Road
Edgbaston
Birmingham
B15 3TN
UK
Telephone: +44 (0)121 300 4345
Email: Fiona.cowdell@bcu.ac.uk
orcid.org/0000-0002-9355-8059

Word count: 5324

Number of figures and tables: one figure and four tables

Keywords: atopic eczema, ethnography, knowledge mobilisation, mindlines, primary care, self-management

Disclaimer and source of support:
This report is independent research arising from a Knowledge Mobilisation Research Fellowship, Professor Fiona Cowdell, KMRF-2015-04-004 supported by the National Institute for Health Research. The views expressed in this publication are those of the authors and not necessarily those of the NHS, the National Institute for Health Research, Health Education England or the Department of Health.

For peer review only - http://bmjopen.bmj.com/site/about/guidelines.xhtml

Clean copy BMJ Open KM Practitioner mindlines R1 15.3.19 2

Knowledge mobilisation: an ethnographic study of the influence of practitioner mindlines on atopic eczema self-management in primary care in the United Kingdom

Abstract

Objective: To explore how atopic eczema specific mindlines are developed by primary care practitioners.

Design: Ethnographic study.

Setting: One large, urban general practice in central England.

Participants: In observation, all practitioners and support staff in the practice and in interviews a diverse group of practitioners (n=16).

Results: Observation of over 250 hours and interview data were combined and analysed using an ethnographic approach through the lenses of mindlines and self-management. Three themes were identified: beliefs about eczema, eczema knowledge and approaches to self-management. Eczema mindlines are set against a backdrop of it being a low priority and not managed as a long-term condition. Practitioners believed that eczema is a simple to manage with little change in treatments available and prescribing limited by local formularies. Practice is largely based on tacit knowledge and experience. Self-management is expected but not often explicitly facilitated. Clinical decisions are made from knowledge accumulated over time. Societal and technological developments have altered the way in which practitioner mindlines are developed; in eczema, for most, they are relatively static.

Conclusions: The outstanding challenge is to find novel, profession and context-specific, simple, pragmatic strategies to revise or modify practitioner mindlines by adding reliable and useful knowledge and by erasing outdated or inaccurate information thus potentially improve quality of eczema care.

Strengths and limitations

- First ethnographic study to examine the development of atopic eczema specific mindlines
- Diverse sample primary care practitioners
- Ethnographer was a lone researcher
- Results may be context specific

For peer review only - http://bmjopen.bmj.com/site/about/guidelines.xhtml

Clean copy BMJ Open KM Practitioner mindlines R1 15.3.19 2

Introduction

Atopic eczema (hereafter “eczema”) is a common, long-term skin condition affecting around one in five children and one in twelve adults in the United Kingdom (UK). It can have a detrimental impact on wellbeing and quality of life and globally is one of the fifty most burdensome diseases (1). Eczema is mainly treated in primary care (2). People may seek advice from general practitioners (GPs), practice nurses (PNs), nurse practitioners (NPs), health visitors (HVs), community pharmacists (CP) and pharmacy counter staff (PCS).

GP consultations are often unsatisfactory for both patient (3) and practitioner (4, 5) with GPs dominating encounters and using avoidance tactics (6) and there being significant dissonance between patient / parent and GP beliefs about assessment and treatment (6). Many GPs have limited specialist dermatology knowledge (7). Nurse consultations, albeit in secondary care, tend to be more positively evaluated (8, 9) and minimal research has been conducted into the contribution of HVs. Research into the role of the CP in dermatology care is limited (10) and expertise may be suboptimal (11) despite CPs reporting being at least reasonably confident in their role (12). The role of the pharmacy counter assistant is equally under-researched although they are often first point of contact for customers and may offer health advice independent of pharmacists (10, 13).

The mainstay of eczema treatment is the regular application of emollients, at least daily and often for many years, with or without intermittent topical steroids and calcineurin inhibitors. Non-adherence results from the high self-management demand of applying topical treatments (14) but also lack of information and conflicting advice from different health professionals (15). Despite available evidence (for example the Global Resource for Eczema Trials database (16) and the National Institute for Health and Care Excellence, Guideline for Eczema (17)) providing evidence-based treatment appears to be a challenge for health professionals managing eczema (18).

For peer review only - http://bmjopen.bmj.com/site/about/guidelines.xhtml

Self-management is a policy imperative which can improve disease outcomes and quality of life for people living with long-term conditions (19). Strategies to support eczema self-management are poorly understood, have limited availability, can be costly and have variable impact (20). Eczema is not classified as a long-term condition in the same way as other illnesses for example asthma (21).

Primary care practitioners are expected to deliver evidence-based practice (EBP). Evidence based medicine (EBM) was originally the preserve of doctors and was defined as "the conscientious, explicit, and judicious use of current best evidence in making decisions about the care of individual patients" (22). Over time, other professions have embraced EBP but this has, at times, been conceptualised as a set of research-based facts which if disseminated to practitioners will ensure more standardised, high quality care (23); this notion is now largely dismissed (24). Primary care practitioners face particular challenges in EBP given the volume of information they need and information overload is a real problem (25).

The study of knowledge mobilisation (KM) is growing exponentially in health care, at its simplest it is "moving knowledge to where it can be most useful" (26). KM involves determined efforts to create, share and use research and other forms of knowledge predicated on the understanding that to be effective KM activity must be relational, constructed from social interaction and context-specific (27-29).

Mindlines, developed from a primary care based ethnographic study (30) offer a "real world" approach to mobilising knowledge and changing clinical practice. Mindlines are "collectively reinforced, internalised tacit guidelines" which underpin clinical decision-making (30). They build on the work of Polyani (31) and Nonaka and Takeuchi (32) who propose that knowledge is not necessarily conscious and explicit, and that tacit knowledge in the form of unconscious schemata and technical know-how are dominant influencers of action compared with formal codified knowledge. Gabbay and LeMay (28) suggest that mindlines are based on flexible, embodied and intersubjective understanding of knowledge that is grounded in the acceptance that there are multiple realties and that knowledge is context-specific. Mindlines represent a complex amalgamation of knowledge gathered from many sources for example, communication with colleagues and

For peer review only - http://bmjopen.bmj.com/site/about/guidelines.xhtml

For peer review only

opinion leaders in the field and from tacit knowledge developed over time (28). In their original work, Gabbay and LeMay (30) examined the construction of mindlines across primary care. A subsequent synthesis of 10 years of mindline literature (n=340) reports that they have been conceptualised and used in four distinct ways. "Nominal" in which the term was used in name only, sometimes with a degree of scepticism, "in practice" examining how mindlines are developed and spread in everyday practice, "theoretical and philosophical" in which the aim was to extend existing theory and "solution focused", exploring ways in which mindlines can be influenced. Solution focused papers (n=28) emphasise the importance of collaborative learning, relationship building and effective leadership in the development of valid, collective, evidence-based mindlines. This review reveals a paucity of information about development or strategies to amend condition specific mindlines (33). Repeating the search strategy utilised for this review in 2018 revealed an abundance of further related literature but little directly addressing condition specific mindlines or how they may best be amended.

Given the prevalence of eczema, the challenges of primary care consultations and the high self-management demand, it is prudent to investigate the way in which eczema mindlines are constructed by practitioners. This will inform understanding of mindlines "in practice" and will underpin future "solution focused" work to develop novel, context-specific, simple and pragmatic strategies to revise or modify eczema mindlines by adding reliable and useful knowledge and by erasing outdated or inaccurate information thus potentially improve quality of eczema care and self-management.

Method

Aim

To understand construction of healthcare practitioner atopic eczema mindlines in primary care.

Design

An ethnographic approach was employed. Ethnography is founded in anthropology and is concerned with the systematic study of people and cultures (34). Data is collected through extensive observation with

For peer review only - http://bmjopen.bmj.com/site/about/guidelines.xhtml

informal conversations, field notes and interviews (35, 36). Data was collected in one large general practice in England.

Setting, participants and process

Data were collected by the author, a nurse and researcher, from January - June 2017. The General Practice was identified by a local clinical research network. It was a research and education active urban general practice in a demographically diverse and deprived area of England with a patient population of approximately 10,000. Observations were also conducted in a community pharmacy adjacent to the practice, which was used by most patients. No practitioners reported a special interest in dermatology. In preparation for data collection the researcher attended two practice meetings to outline conduct of the study. Data were collected in more than 250 hours of observation during all surgery opening hours. The role of social-participant-as-observer, that is, predominantly observer with some social functions such as cleaning couches was taken (37). Observation began with the reception team to understand the day-to-day working of the practice. Observation of consultations with GPs, GP trainees and locums, nurses, health visitors in baby clinics, held on the practice premises, and pharmacy staff followed. GP telephone consultations were listened to and discussed with the practitioner. Field notes were documented and informal conversations either written contemporaneously or audio-recorded. Entire clinics were attended regardless of presenting complaint, to gain understanding in the context of other long-term conditions. Between consultations practitioners recounted recent eczema consultations. Available documentation was reviewed. Single, semi-structured interviews using a topic guide (Table 1) were conducted with practitioners from each profession (n=16) (Table 2) using maximum variation purposive sampling (38) to ensure a mix of job role and level of experience. A predominance of female participants was reflective of the profile of the healthcare team. The complete dataset is summarised in Table 3.

Table 1: Practitioner interview topic guide

- Do you have any special interest in skin health?
- How much contact do you have with patients with eczema?

For peer review only - http://bmjopen.bmj.com/site/about/guidelines.xhtml

Clean copy BMJ Open KM Practitioner mindlines R1 15.3.19 2

- What sort of treatments do you use most often?
- How do you decide on a particular treatment?
  - What impact does the local formulary have on your prescribing?
- How much are you able to advise patients on how to care for their eczema?
  - Concordance etc
- How do you update your own knowledge about eczema?
- How could we best get research information to use in your practice?
  - What methods do you use now?
  - Can you give any specific examples?
- Do patients come with their own ideas about the treatment they need?
- How much do you and your patient share the decision about what treatment to use?
- How do you reconcile patient's needs with what is available?
- Do you refer patients to any external sources of information?

Table 2: Demographic details of interview participants

**Role**	**Gender**	**Years in current role**
Health visitor	Female	10
GP	Male	35
GP Trainee	Female	2
Practice nurse	Female	31
Practice nurse	Female	32
Pharmacist	Male	8
GP Trainee	Female	5
Pharmacist	Female	12
Pharmacy counter staff	Female	10
Pharmacy counter staff	Female	17

For peer review only - http://bmjopen.bmj.com/site/about/guidelines.xhtml

Clean copy BMJ Open KM Practitioner mindlines R1 15.3.19 2

GP Trainee	Female	7
GP	Female	6
GP	Female	5
Health visitor	Female	2
Health visitor	Female	2
Health visitor	Female	3

Table 3: Complete dataset

**Observations and informal interviews**
1 General practice
10 sessions observing reception and waiting room
9 sessions observing in baby clinics
2 sessions observing in community pharmacy
24 sessions observing GPs
5 sessions with practice manager
Multiple informal meetings and one to one informal discussions
4 practice meetings
6 debriefs with GP trainees
**Formal interviews**
16, details provided in table 2
**Documentary sources**
Local prescribing guidelines
Online guidance accessed by practitioners during observation

Interviews were conducted in the workplace and lasted from 22-40 minutes. Data sufficiency was achieved when no new insights were forthcoming (39). For completeness documents and websites were reviewed

For peer review only - http://bmjopen.bmj.com/site/about/guidelines.xhtml

including the NICE Clinical Guideline for Eczema (17), the local emollient formulary and the Clinical Knowledge Summary (40) and GP notebook pages (41) for eczema.

Data collection and analysis were iterative with initial findings being used to guide further collection (42). Audio-data were professionally transcribed and transcripts read against the recording by the researcher to confirm accuracy. Data analysis was completed independently by the researcher, though the lenses of mindlines and self-management. Transcripts and field notes were read in full to get a sense of the data as a whole, and then manually coded, categorised and merged into themes and annotated with researcher inductive interpretations (see table 4 for worked example). Post theme development, relevant sections of the data were revisited to ensure authentic interpretation and use of participant language.

Table 4: Example of data analysis process

**Codes (from interview and observational data)**	**Categories**	**Theme**
GP interview<br>• Eczema "simple to treat" nothing much has changed over the years – it's bread and butter to us	It's simple to treat	Beliefs about eczema
HV interview<br>• Basics are the same, but there's lots of personal preference		
GP interview<br>• Common complaint "know by heart"		
Observational data<br>• Perception from GPs that it's a straightforward condition, treatment is fairly standard and that there is limited need for further knowledge. Intranet rarely used but fairly standard set of resources for GPs		
GP interview<br>• Software will fire up a message if another product should be used	No need to think too much	
Pharmacist interview<br>• Script Switch – computer tells you if you are prescribing the wrong thing and suggests an alternative		
Observational data<br>• Belief that guidance is more about cost that research		
Observational data<br>• Eczema is not a condition that is mentioned in "learning" interactions such as debriefs		

Reflexivity

Reflexivity was maintained throughout the study with particular attention being paid to subjectivity and positioning as a nurse and skin health researcher; pre-understandings were consciously set aside (43).

For peer review only

For peer review only - http://bmjopen.bmj.com/site/about/guidelines.xhtml

Patient and Public Involvement

Lay people, from an eczema support group, were involved in the development of the research question and in planning the design of the study. They contributed through one meeting and a series of email exchanges.

Results

Data analysis resulted in three themes: beliefs about eczema; eczema knowledge and approaches to self-management. Each is discussed with examples from the data below.

Theme 1: Beliefs about eczema

Eczema was consistently viewed as a *"bread and butter"* [GP] condition that accounted for many consultations. However, although 19.5% of the practice population was recorded as having some type of eczema few consultations primarily for this condition were observed. Analysis of patient reported reason for GP consultation for a typical week during observation revealed that 26/627 (4.1%) of reasons were skin related with none citing eczema as the primary complaint. No observed face-to-face consultations were primarily for eczema; it was reported as a secondary concern in a small number of number of GP consultations and more often to HVs in baby clinics. This resulted in eczema necessarily being given limited attention *"it's often a secondary problem and there's only time to deal with one problem per consultation"* [GP]. Telephone consultations with GPs were witnessed and patients were observed to consult with pharmacy staff about their eczema. Practitioners mainly viewed eczema as a nuisance condition requiring limited knowledge to treat effectively, *"eczema is simple to treat, nothing much has changed over the years"* [GP] and *"the recipe doesn't change* [GP]".

Some GPs described eczema as a *"catch up"* [GP] consultation when clinics were over-running. GPs and nurses noted the absence of specific external incentives for long-term eczema management and that it was a condition without the *"red flags"* [GP] which trigger treatment escalation or referral. They described treatment options as straightforward involving emollients with or without intermittent topical steroids.

For peer review only - http://bmjopen.bmj.com/site/about/guidelines.xhtml

Few mentioned calcineurin inhibitors or other available medications. Most practitioners considered emollients to be a homogenous group of preparations all with similar properties, although a few differentiated in terms of viscosity and texture. Pharmacy staff and HVs were familiar with a broader range of emollient products and were more likely to offer suggestions for over the counter preparations. This was in part because no HVs in this study were able to prescribe. GPs were reluctant to prescribe topical steroids or other treatments unless absolutely necessary. PNs rarely saw patients with atopic eczema.

Practitioners recognised that eczema could have a negative impact on wellbeing and quality of life but this was not often reflected in the care offered. Treatment was mainly in reaction to a flare rather than there being a long-term plan of care. Generally patients were able to access regular repeat prescriptions for emollients and practitioners expressed a level of frustration when they presented with a flare having not requested or used the prescribed treatments. Although 'safety netting' was always in place, planned follow-up consultations were not suggested. Empathy for patients was most evident in practitioners who had personal experience of eczema, they articulated a varying level of understanding about the differences between products, regardless of available empirical evidence, and the extent to which personal preference influenced concordance. Pharmacy counter staff were the most conversant with the differences between emollient products having tried samples, and they were most likely to share this knowledge with patients / customers verbally and in leaflets.

Although eczema was viewed as a frequent reason for consultation, it was mainly presented as a secondary concern and so dealt with swiftly. Eczema was considered simple to treat with little change over time although practitioners with personal experience of eczema were more aware of the challenges of self-management and tolerant of personal treatment preferences.

Theme 2: Atopic eczema knowledge

Beliefs about eczema influenced the formation of mindlines and for most mindlines were set against a backdrop of eczema being a low priority condition and a perception of unchanging treatment options which

For peer review only - http://bmjopen.bmj.com/site/about/guidelines.xhtml

were constrained by local prescribing guidelines (Figure 1). Many practitioners described AE as a common conditions for which you "*know [treatment] by heart* [GP]" and likened his response to using a "*satnav ………..you stop thinking, the little NHS boxes [on the computer] tell you what to prescribe* [GP]".

Most practitioners reported that their AE knowledge was based on their initial education and recognised "*pre-reg derm education was very, very basic"* [GP]. A few had completed dermatology placements during GP training but reported seeing little AE. One experienced GP recounted learning from a consultant, her practice was unchanged as she had "*learnt from a consultant many years ago and never heard anything to contradict it"* [GP]. PNs and GPs were aware of available dermatology education but did not attend as it was a low priority and costly, "*there is training but you have to pay"* [PN] and they preferred to "*avoid reps and sponsored sessions"* [GP]. HVs reported that skin health was never an educational priority. Pharmacist's knowledge was updated through e-bulletins from different sources and covered only changes in, and availability of, medications. Only PCS received eczema specific education by attending regular seasonal sessions provided by their employer. Although deemed to be useful, particularly as they tried products and were advised on correct application, the educational experience was sometimes suboptimal as one reported how she was "*shamed into remembering"* [PCS] session content.

Local emollient guidelines underpinned many prescribing decisions so practitioners did not need to think as "*software will fire up a message if another product should be used"* [CP]. Changes to guidelines were ascribed to cost and "*what was in vogue"* [GP]. Practitioners were not concerned about these changes stating for example, "*aqueous cream, they've gone off that idea for some reason"* [PCS] and "*Zero products are the ones that are currently on trend"* [GP trainee]. Whilst some prescribers stuck rigidly to prescribing the cheapest product, "*I try to be good and prescribe the cheaper side of things"* [GP trainee], others were more flexible according to their own or the patient's preference. However, deviations from the formulary were rare on the basis that "*local formulary is very constraining and you'd have to be able to justify why you'd prescribed anything else"* [GP]. Exceptions were observed in the baby clinic and in pharmacy practice where patients were often informed about a wider range of emollients that could be purchased over the

For peer review only - http://bmjopen.bmj.com/site/about/guidelines.xhtml

counter. For those who paid a prescription charge this could often be more cost effective. PCS suggested that they were able to advise patients readily as they had "*tried samples so you can tell the customers what they feel like*" [PCS].

Other knowledge sources contributed to eczema mindlines. All staff, with the exception of experienced GPs, used internet searches most commonly the online resources GP Notebook and Clinical Knowledge Summaries. Useful websites were often book-marked and visited in preparation for a consultation rather than alongside the patient. If information was not located almost immediately the practitioner switched to another website "*we're hard wired for speed now*" [GP] and "*dipped into what's relevant*" [GP] as and when required. None mentioned existing NICE Eczema Guidelines. Local emollient guidelines existed and influenced the prescribing practice of most practitioners, however others were unaware of these and some found them hard to access. GPs and HVs used different emollient guidelines and this caused confusion for patients when they consulted both. A member of pharmacy staff noted the need for "*a synchronised approach so patients don't get confused*" [PCS]. Practitioners also experienced confusion when offering advice on treatment application, for example "*treatment is a bit arbitrary – for example should you advise steroid or emollient first?*" [HV].

Practitioners learnt from each other to a limited extent, most often within their professional groups. They recognised "*we learn both good and bad habits from each other*" [PN]. Opportunities for shared learning had reduced as there was little time to meet up and in-house teaching for GPs and PNs had "*fallen by the wayside*" [GP] due to staff sickness and pressure of work. One GP reported "*phoning a friend*", now a consultant dermatologist, when she needed advice. GPs reported learning from trainees during debrief sessions but could not recall ever having discussed eczema. Trainees exhausted all available information sources before seeking advice from a GP. HVs and PNs met more frequently and exchanged knowledge more regularly, although eczema was not a condition of interest.

For peer review only - http://bmjopen.bmj.com/site/about/guidelines.xhtml

Practitioners expressed varied views on the value of patient knowledge and experience and the extent to which it influenced care. PNs, HVs and pharmacy staff respectively reported that they routinely "*ask patient what they have tried already*" [PN], "*see what's worked for them*" [HV] and "*listen and learn from customers*" [PCS] and used this information as a basis for treatment advice. Others listened to patients with a degree of scepticism but acquiesced to patient preference, "*patients often have fixed ideas [about emollients] and I try to accommodate these*" [GP]. A few were less receptive, for example "*I try to use guidelines and the formulary ………… patient experience stuff can be counterproductive*" [GP trainee] and others suggested that their wider experience overrode the patients personal preferences and experiences "*experience wise I've found a lot of people get on with it [particular emollient]*" [GP] and therefore that was what would be prescribed.

Only the most experienced practitioners spontaneously articulated the existence of tacit knowledge stating, "*it's a perpetual exercise … adding on knowledge and skills*" [GP] and "*built up knowledge over time*" [PN]. Others pointed to more concrete sources of knowledge. All practitioners understood reliability of evidence to a greater or lesser extent.

Eczema knowledge was constructed from different sources by individual professions. Nursing and medical staff perceived a limited need to update their knowledge as eczema care was viewed as having changed little over time. Exceptions to this were practitioners who had personal experience of eczema and pharmacy staff who regularly updated their mindlines using informal and formal sources of knowledge.

Theme 3: Approaches to self-management

In principle, all practitioners supported self-management of eczema but recognised the difficulties of achieving this in practice particularly without formal recognition as a long-term condition (LTC). Some practitioners routinely used techniques to support self-management for patients with other LTCs. Strategies included for example by "*finding out patients' expectations*" [PN], "*tailoring knowledge to the person*" [GP], "*start with what the patient understands and then fill in the gaps*" [GP], "*give patients a map*

For peer review only - http://bmjopen.bmj.com/site/about/guidelines.xhtml

of management" [GP], "*instil confidence"* [GP] and "*reinforce that self-management is good"* [GP trainee]. A few GPs used specific techniques such as "*short bursts of CBT"* [GP], "*motivational interviewing techniques ……. compressed to fit in consultation"* [GP] and "*behaviour modification ……….. not a one consultation job"* [GP]. Even practitioners who did not articulate using strategies to support self-management integrated them in practice for many LTCs. However they were rarely observed or discussed in relation to eczema.

Most eczema care was reactive when patients presented with a flare and talk of eczema care was almost exclusively about treatment options with virtually no attention paid to ensuring that the patient understood the condition and actions they could take to avoid the relentless cycle of flares. The most tangible contribution to self-management was the availability of repeat prescriptions for emollients but advice to use these consistently was lacking. Barriers to self-management were observed, for example the appointment system often precluded patients seeing the same GP over time so treatment could be altered without the benefit of fully understanding the patient journey to date. Contradictory advice given by practitioners and a lack of faith in patient's ability to judge when they needed to use topical steroids and to use them safely presented significant barriers to successful self-management. Practitioners suggested the "*need to see patients before prescribing [topical] steroids"* [GP], one GP stated that "*sensible*" patients may get steroids on repeat but struggled to quantify sensible in this context. Pharmacy staff did not recognise their contribution to self-management per se but recognised the positive impact they had on eczema management through "*actually taking notice of what they're telling me"* [PCS] and perceived "*they do trust me ………………. I'm well known in the local community"* [PCS] and were therefore easy for customers to speak with.

Whilst recognising the need for self-management the fact that eczema is not categorised as a long-term condition limited how much patients were supported to self-manage and at times healthcare systems could hinder attempts.

For peer review only - http://bmjopen.bmj.com/site/about/guidelines.xhtml

Discussion

This study offers new insights into how primary care practitioners construct atopic eczema specific mindlines. Practitioner mindlines are predominantly set against a back drop of eczema being a low priority, due to a combination of not being viewed as an LTC and so lacking external incentives and the perception of available treatments being standard use of emollients and topical steroids, which changes little over time and is constrained by prescribing guidance. This led to an assumption that there was little need to amend mindlines. Eczema mindlines were developed early in their career by many practitioners and were relatively static amongst GPs, PNs and HVs, except for those with direct personal experience of eczema. Mindlines of pharmacy staff were regularly modified through a combination of education provided by their employer, electronic updates from professional bodies and interactions with customers. The latter was particularly influential for the PCSs as they generally had more time to listen and had built up trusting relationship with the customers over time.

This study is one of few to apply mindline theory to a specific condition across a broad range of practitioners. In particular it identifies important differences in the way in which eczema mindlines are developed and so may best be amended for individual practitioner groups. This study conforms with conventions of robust qualitative work in that it is rigorous (coherent and sufficiently well reported to be open to external audit), relevant (enriches understanding of the subject), resonant (resonates with readers experiences and understandings) and reflexive (subjectivity of the author is acknowledged) (44). Limitations include issues of reliability as the ethnographer is a lone worker and data analysis was completed by the researcher alone, however this is mitigated by conversations with participants to check understandings. As data was collected in one general practice, findings may not be transferable but the diversity of participants should minimise this risk (45). Additionally no nurse practitioners were included as, at the time of data collection, none were employed in the practice.

As with the original conceptualisation of Gabbay and leMay (30), practitioner eczema mindlines are composed over time, from a range of evidence sources which rarely embrace direct use of research.

Gabbay and le May (28) point to the critical nature of knowledge-in-practice-in-context in which in each context new knowledge is converted by the complex social processes of the Socialization, Externalization, Combination, Internalization spiral (32). Context was central in the formation of eczema mindlines but was informed more by long-held beliefs and national policy than by local context. Key differences in this study are that mindline development has evolved alongside the changing nature of primary care where practitioners, particularly GPs, appear to work more in isolation than as part of a community with "coffee room chat" (46) appearing much reduced. In parallel available online resources have spiralled thus potentially reducing the need to confer with others. This challenges the notion that mindlines are heavily reliant on professional interactions (28). The static nature of eczema mindlines and the beliefs underpinning eczema care meant that they were accessed using fast, automatic, System 1 thinking rather than the more deliberative, conscious slow and effortful System 2 approach (47).

Few studies have investigated condition specific mindlines with the exception of a Tanzanian study of malaria diagnosis (48), however the depiction here is more akin to rules of thumb or heuristics. A comprehensive commentary on mindlines identifies 76 papers categorised as "in practice", that is studies of how mindlines are developed, many of these used the term to mean consulting with colleagues (33). A smaller number were faithful to the original Gabbay and Le May's conceptualisation but add little by way of new understanding. More recently Wieringa and colleagues (49) investigated mindlines development in online clinical communities concluding that they offered collective, dynamic settings and implicitly that they may be areas for mindline amendment. Whilst online communities may appeal to some practitioners, this will not be so for all.

In this study eczema was consider low priority. These beliefs are longstanding with surveys suggesting that both patients and practitioners perceive dermatology as a poor relation in healthcare (50-52) and Magin and colleagues (4) describing 'dismissive' and 'unsympathetic' attitudes amongst GPs. Eczema appears to be considered as "health problem which is not an illness" (53) and therefore less legitimate and worthy than other conditions. Ambivalence about eczema specific learning was in contrast to a survey which

For peer review only - http://bmjopen.bmj.com/site/about/guidelines.xhtml

indicated a desire for new knowledge, particularly in the form of education delivered by consultants (54); inevitably GPs completing the survey would be those with an interest in dermatology. The dermatology community has used many strategies to make research findings accessible to all with limited success (55). In contrast with this study in which treatment for eczema was viewed as simple others report GPs uncertainty about managing eczema (56).

Achieving change in primary care practice is challenging, interventions most likely to influence practice demonstrate evidence of benefit, are simple to use and adaptable to local context (57). The context of eczema mindlines, that it is a low priority condition with a limited repertoire of treatment options, is unlikely to change in the foreseeable future. If, like other LTCs eczema was recognised in the Quality and Outcomes Framework (58) patients may benefit from the accelerated trends towards systematic management (59). Practitioners in primary care are expert generalists (60) and are expected to have knowledge of many conditions for which there is wealth of available evidence. This may lead to information overload for which coping strategies are needed. Bate and colleagues (61) describe "satisficing" that is, curtailing the amount of information gathered to enable them to make a "good enough" decision.

In many ways it can be argued that treatment of eczema in primary care is relatively straightforward and that amendment of mindlines to adjust thinking about emollients and removal of outdated information about topical steroid use could make a significant change in practice that would improve both patient experience and self-management practices. Brevity and accessibility of information is key as practitioners have been found to judge the usefulness of new knowledge as function of its relevance x validity ÷ by the work needed to access it (62). It is possible that straightforward messages could be conveyed through media such as aphorisms, "succinct sayings that offer advice" (63) or actionable nuggets "knowledge translation tools designed to provide …….. concise practical information about the most prevalent and pressing primary care needs of patients" (64). This approach offers the opportunity to compensate for the loss of professional wisdom through personal communication by transmitting concentrated wisdom and guidance in a different way (63).

For peer review only - http://bmjopen.bmj.com/site/about/guidelines.xhtml

Clean copy BMJ Open KM Practitioner mindlines R1 15.3.19 2

Efforts to amend GPs, PNs and HVs mindlines need to be accessible via rapid System 1 thinking. Interventions should be specific, practical, tailored, relevant and rapidly delivered information which can readily be assimilated, or as participants in this study described it, a “no faff” approach. Given their time constraints and information gathering habits any new information would best be delivered individually rather than in a group setting and available online and possibly in other formats.

The role of the community pharmacist in eczema care is evolving partly in response to Pharmaceutical Services Negotiating Committee guidance on Medicines Use Reviews (65), New Medicine Service (66) and Minor Ailment Service (67). Forthcoming changes in availability of emollients on prescription may increase their role further. Pharmacy staff described eczema mindline development as a more collective experience than other practitioners and valued learning from each other and from customers. They may be open to group approaches to update and remove redundant information from their mindlines and this would need to be brokered through both professional and employing organisations.

Conclusion

This ethnographic study provides new understandings about the development of atopic eczema specific mindlines in different practitioner groups in primary care. The outstanding challenge is to find novel, context-specific, simple, pragmatic strategies to revise or modify these mindlines by adding reliable and useful knowledge and by erasing outdated or inaccurate information using strategies that are most appropriate to each profession. Mindline amendment has the potential to improve self-management and quality of eczema care through the delivery of consistent, evidence-based care.

For peer review only - http://bmjopen.bmj.com/site/about/guidelines.xhtml

Clean copy BMJ Open KM Practitioner mindlines R1 15.3.19 2

Contribution statement

FC is the sole contributor to this paper

Funding

This report is independent research arising from a Knowledge Mobilisation Research Fellowship, Professor Fiona Cowdell, KMRF-2015-04-004 supported by the National Institute for Health Research. The views expressed in this publication are those of the authors and not necessarily those of the NHS, the National Institute for Health Research, Health Education England or the Department of Health.

Ethical approval

The study was approved by a National Health Service REC (16/YH/0252). Process consent was used for observation, on each occasion informal conversations were used to re-check participant's willingness to be observed. Patients were informed about the study by practitioners and when necessary the researcher exited individual consultations, either at the request of the patient, the practitioner or using personal judgment, although this was infrequently needed. Written consent was taken for audio-recorded interviews. Interview participants consented to publication of anonymised information.

Competing interests

None to declare

Acknowledgements

Thanks to Amanda Roberts who have given invaluable lay feedback on the planning and design of this study. Thanks to James Mycock, Birmingham City University for the mindline illustration and to Professors Hywel William and Stephen Timmons, University of Nottingham for their valuable feedback on earlier iterations of this manuscript

Data sharing statement

The datasets generated and/or analysed during the current study are not publicly available as they are not designed to be re-analysed by others but are available from the corresponding author on reasonable request.

For peer review only

Clean copy BMJ Open KM Practitioner mindlines R1 15.3.19 2

References

1. Hay RJ, Johns NE, Williams HC, et al. The global burden of skin disease in 2010: an analysis of the prevalence and impact of skin conditions. J Invest Dermatol. 2014; 134: 1527-1534.
2. Schofield J, Grindlay D, Williams H, Skin conditions in the UK: a health care needs assessment. Centre of Evidence Based Dermatology, University of Nottingham; 2009.
3. Cowdell F. Knowledge mobilisation: an ethnographic study of the influence of lay mindlines on eczema self-management in primary care in the United Kingdom. BMJ Open. In press
4. Magin P, Adams J, Heading G, Pond C. Patients with skin disease and their relationships with their doctors: a qualitative study of patients with acne, psoriasis and eczema. Med J Aust. 2009; 190:62-64
5. Santer M, Burgess H, Yardley L, et al. Experiences of carers managing childhood eczema and their views on its treatment: A qualitative study. Br J Gen Pract. 2012; 62:261-267.
6. Powell K, Le Roux E, Banks J, et al. GP and parent dissonance about the assessment and treatment of childhood eczema in primary care: a qualitative study. BMJ Open. 2018; 8:e019633.
7. Rübsam M, Esch M, Baum E, et al. Diagnosing skin disease in primary care: A qualitative study of GPs' approaches. Fam Pract. 2015; 32: 591-595.
8. Nicol NH, Ersser SJ. The role of the nurse educator in managing atopic dermatitis. Immunol Allergy Clin. 2010; 30:369-383.
9. Rolinck-Werninghaus C, Trentmann M, Reich A, et al. Improved management of childhood atopic dermatitis after individually tailored nurse consultations: a pilot study. Pediat Allergy Immunol. 2015; 26: 805-810.
10. Tucker R, Stewart D. Why people seek advice from community pharmacies about skin problems. Int J Pharm Pract. 2015; 23(2): 150-153.
11. Tucker RP, MacLure K, Paudyal V, et al. An exploratory study of community pharmacist diagnosis and management of dermatitis and acne. Selfcare J. 2017; 8(2):1-10
12. Tucker R. The medicines use review in patients with chronic skin diseases: are pharmacists doing them and how confident are they? Int J Pharm Pract. 2013; 21:202-204.
13. Banks J, Shaw A, Weiss MC. The community pharmacy and discursive complexity: a qualitative study of interaction between counter assistants and customers. Health Soc Care Community. 2007; 15:313-321.
14. Santer M, Burgess H, Yardley L, et al. Managing childhood eczema: Qualitative study exploring carers' experiences of barriers and facilitators to treatment adherence. J Adv Nurs. *2013*; 69:2493-2501.
15. Teasdale EJ, Muller I, Santer M. 2017. Carers' views of topical corticosteroid use in childhood eczema: a qualitative study of online discussion forums. Br J Dermatol. 2017; 176(6):1500-1507.
16. Global Resource for Eczema Trials (GREAT) database http://www.greatdatabase.org.uk/GD4/Home/Index.php Accessed 4.7.18
17. National Institute for Health and Care Excellence. Atopic eczema in under 12s: diagnosis and management. Clinical guideline [CG57] 2007 https://www.nice.org.uk/Guidance/CG57. Accessed 4.7.18
18. Powell K, Le Roux E, Banks JP, et al. Developing a written action plan for children with eczema: A qualitative study. Brit J Gen Pract. 2018; 68(667):e81-e89.
19. Department of Health 2014 The Mandate: A mandate from the Government to NHS England: April 2014 – March 2015 https://www.gov.uk/government/uploads/system/uploads/attachment_data/file/383495/2902896_DoH_Mandate_Accessible_v0.2.pdf Accessed 4.7.18
20. Ridd MJ, King AJL, Le Roux E, et al. Systematic review of self-management interventions for people with eczema. Br J Dermatol. 2017; 177(3):719-734.
21. Hanlon P, Daines L, Campbell C. et al. Telehealth interventions to support self-management of long-term conditions: a systematic metareview of diabetes, heart failure, asthma, chronic obstructive pulmonary disease, and cancer. JMIR. 2017; 19:e172.

For peer review only - http://bmjopen.bmj.com/site/about/guidelines.xhtml

22. Sackett, DL, Rosenberg WM, Gray JM, et al. Evidence based medicine: what it is and what it isn't. BMJ. 1996; 312:71-2.
23. Greenhalgh T, Howick J, Maskrey N. "Evidence based medicine: a movement in crisis?" BMJ 348 (2014): g3725.
24. Brownson RC, Colditz GA, Proctor EK. eds. Dissemination and implementation research in health: translating science to practice. Oxford: Oxford University Press; 2017.
25. Vaucher C, Bovet E, Bengough T, et al. Meeting physicians' needs: a bottom-up approach for improving the implementation of medical knowledge into practice. Health Res Policy Syst. 2016; 14:49.
26. Ward V. 2017. Why, whose, what and how? A framework for knowledge mobilisers. Evid Policy. 2017; 13:477-497.
27. Ferlie E, Crilly T, Jashapara A, et al. Knowledge mobilisation in healthcare: a critical review of health sector and generic management literature. Soc Sci Med. 2012;74:1297-1304.
28. Gabbay J, Le May A. Practice-based evidence for healthcare: clinical mindlines. London: Routledge; 2010.
29. Plamondon K, Caxaj S. 2018. Toward Relational Practices for Enabling Knowledge-to-action in Health Systems: The Example of Deliberative Dialogue. Adv Nurs Sci. 2018; 41:18-29.
30. Gabbay J, le May A. Evidence based guidelines or collectively constructed "mindlines?" Ethnographic study of knowledge management in primary care. BMJ. 2004; 329:1013.
31. Polanyi M. The logic of tacit inference. Philos.1966; 41:1-18.
32. Nonaka I, Takeuchi H. The knowledge-creating company: How Japanese companies create the dynamics of innovation. Oxford: Oxford University Press; 1995.
33. Wieringa S, Greenhalgh T. 10 years of mindlines: a systematic review and commentary. Imp Sci. 2015; 10:45.
34. Hammersley M, Atkinson P. Ethnography: Principles in Practice. Abingdon: Routledge;2007.
35. Spradley JP. Participant observation. Illinois: Waveland Press; 2016.
36. Spradley JP. The ethnographic interview. Illinois: Waveland Press; 2016.
37. Cowdell F. That's how we do it we treat them all the same: An exploration of the experiences of patients, lay carers and health and social care staff of the care of people with dementia in acute hospital settings. Cambridge. Cambridge Publishing. 2013.
38. Tongco MD. Purposive sampling as a tool for informant selection. Ethnobotany Research and Applications. 2007. 31; 5:147-58.
39. O'Reilly M, Parker N. 'Unsatisfactory Saturation': a critical exploration of the notion of saturated sample sizes in qualitative research. Qual Res. 2013; 13:190-197.
40. Clinical Knowledge Summaries. Eczema – atopic https://cks.nice.org.uk/eczema-atopic Accessed 4.7.18
41. GP Notebook. Eczema http://www.gpnotebook.co.uk/simplepage.cfm?ID=-623902719 Accessed 4.7.18
42. Polkinghorne DE. Language and meaning: Data collection in qualitative research. J Couns Psychol, 2005; *52*(2):137.
43. Matua GA, Van Der Wal DM. Differentiating between descriptive and interpretive phenomenological research approaches. Nur Res. 2015; 22(6):22.
44. Finlay L. Phenomenology for psychotherapists: Researching the lived world. Oxford: Wiley Blackwell; 2011.
45. University of Hertfordshire 2017 Disadvantages of Ethnographic Research http://www.health.herts.ac.uk/immunology/Web%20programme%20-%20Researchhealthprofessionals/disadvantages_associated_with_et.htm. Accessed 4.7.18
46. Gabbay J, le May. Mindlines: making sense of evidence in practice. B J Gen Pract. 2016: 402-403.
47. Kahneman D. Thinking, Fast and Slow. London: Penguin; 2011.
48. Chandler CI, Jones C, Boniface G et al. Guidelines and mindlines: why do clinical staff over-dignose malaria in Tanzania? A qualitative study. Malar J 2008;7:53
49. Wieringa S, Engebretsen E, Heggen K, et al. How knowledge is constructed and exchanged in virtual communities of physicians: qualitative study of mindlines online. JMIR. 2018;20: e34.

For peer review only - http://bmjopen.bmj.com/site/about/guidelines.xhtml

50. Rosoff SM, Leone MC. (1989). The prestige of dermatologists. are they "last among equals"?Int J Dermatol. 1989; 28:377-380.
51. Rosoff SM, Leone MC (1991). The public prestige of medical specialties: Overviews and undercurrents. Soc Sci Med. 1991;32:321-326.
52. Creed PA, Searle J, Rogers ME. Medical specialty prestige and lifestyle preferences for medical students. Soc Sci Med. 2010;71:1084-8.
53. Cornwell J. Hard-earned lives: accounts of health and illness from East London:Routledge Kegan & Paul;1984.
54. Soriano LF, Sahota A, Jolliffe V. An evaluation of general practitioners learning preferences in dermatology. Education for Primary Care. 2017; 17:1-2.
55. Baron SE, Cohen SN, Archer CB. Guidance on the diagnosis and clinical management of atopic eczema. Clin Exp Dermatol. 2012; 37:Suppl 1, 7-12.
56. Le Roux, E, Powell K, Banks JP, et al. GPs' experiences of diagnosing and managing childhood eczema: a qualitative study in primary care. Br J Gen Pract. 2018. Feb 16:bjgp18X694529.
57. Lau R, Stevenson F, Ong BN, et al. Achieving change in primary care—causes of the evidence to practice gap: systematic reviews of reviews. Imp Sci. 2015;11:40.
58. National Institute for Health and Care Excellence (2018) NICE Quality and Outcomes Framework indicator https://www.nice.org.uk/standards-and-indicators/qofindicators Accessed 4.7.18
59. Roland M, Guthrie B. Quality and Outcomes Framework: what have we learnt? BMJ 2016;354:i4060
60. Reeve J, Dowrick CF, Freeman GK, et al. Examining the practice of generalist expertise: a qualitative study identifying constraints and solutions. JRSM short reports. 2013; 4(12):2042533313510155.
61. Bate L, Hutchinson A, Underhill J, et al. How clinical decisions are made. Br J Clin Pharmacol. 2012; 74:614-20.
62. Cunningham M, Shirley A. Mindlines in a digital age. Education for Primary Care. 2015; 26:293-6.
63. Levine D, Bleakley A. Maximising medicine through aphorisms. Med Educ. 2012; 46:153-162
64. McColl MA, Aiken A, Smith K, et al. Actionable nuggets: knowledge translation tool for the needs of patients with spinal cord injury. Can Fam Physician. 2015; 61:e240-e248.
65. Pharmaceutical Services Negotiating Committee Medicines Use Review http://psnc.org.uk/services-commissioning/advanced-services/murs/ Accessed 4.7.18
66. Pharmaceutical Services Negotiating Committee New Medicine Service http://psnc.org.uk/services-commissioning/advanced-services/nms/ Accessed 4.7.18
67. Pharmaceutical Services Negotiating Committee Minor Ailment Service http://psnc.org.uk/services-commissioning/locally-commissioned-services/en8-minor-ailments-service/ Accessed 4.7.18

For peer review only - http://bmjopen.bmj.com/site/about/guidelines.xhtml

Clean copy BMJ Open KM Practitioner mindlines R1 15.3.19 2

Table 1: Practitioner interview topic guide

Table 2: Demographic details of participants

Table 3: Complete dataset

Table 4: Example of data analysis process

Figure 1: Practitioner eczema mindlines

Sources of information underpinning practitioner eczema mindlines.

For peer review only

For peer review only - http://bmjopen.bmj.com/site/about/guidelines.xhtml

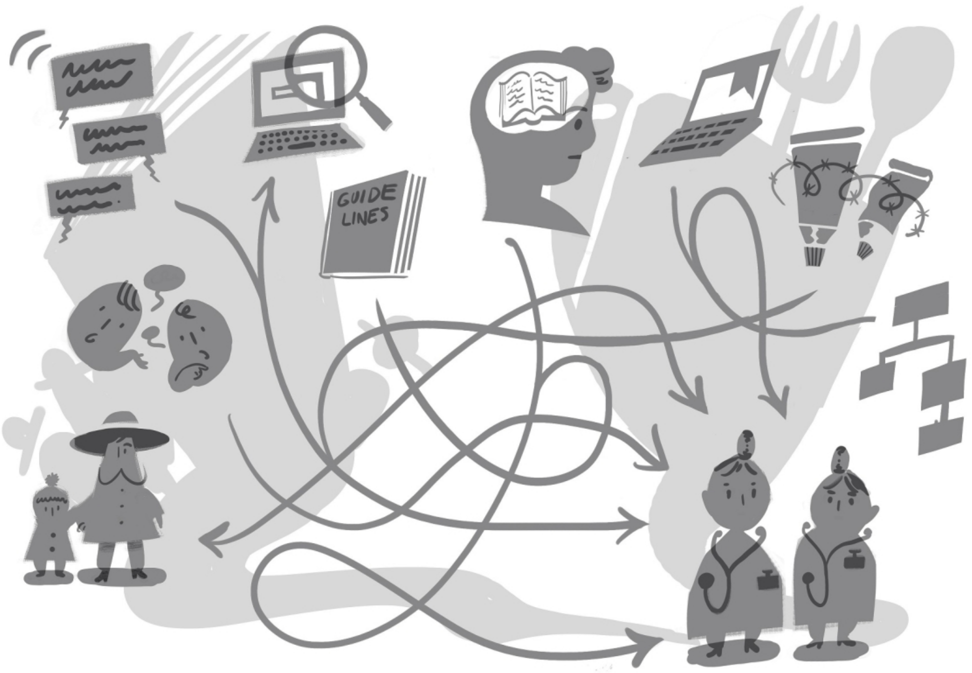

Practitioner eczema mindline

371x262mm (120 x 120 DPI)

BMJ Open Practitioner eczema mindlines COREQ 4.7.18

COREQ Statement

Knowledge mobilisation: An ethnographic study of the influence of practitioner mindlines on eczema self-management in primary care in the United Kingdom

Statement	Page no
**Domain 1: Research team and reflexivity**<br>*Personal Characteristics*<br>1. Interviewer/facilitator Which author/s conducted the interview or focus group?<br>I conducted all observation and interviews	6
2. Credentials What were the researcher's credentials? E.g. PhD, MD<br>DProf, RN	Title page
3. Occupation What was their occupation at the time of the study?<br>Professor of Nursing and Health Research	Title page
4. Gender Was the researcher male or female?<br>Female	Title page
5. Experience and training What experience or training did the researcher have?<br>I have extensive experience in qualitative research.	Title page
Relationship with participants<br>6. Relationship established Was a relationship established prior to study commencement?<br>I established rapport with each observational participant at each meeting and with interviewees at the time of interview.	6
7. Participant knowledge of the interviewer<br>What did the participants know about the researcher? e.g. personal goals, reasons for doing the research<br>I met the team prior to observation, provided an information sheet, explained about myself and the study and answered questions. I gave interview participants an information sheet prior to interviews and answered any questions, I reiterated this information at the beginning of each interview.	6
8. Interviewer characteristics What characteristics were reported about the interviewer/facilitator? e.g. Bias, assumptions, reasons and interests in the research topic<br>Participants were aware, and it is reported in the manuscript, that I am a Registered Nurse with an interest in how eczema knowledge is developed and shared between patients and practitioners in primary care and that this was a publically funded study.	8
**Domain 2: study design**<br>*Theoretical framework*<br>9. Methodological orientation and theory<br>What methodological orientation was stated to underpin the study? e.g. grounded theory, discourse analysis, ethnography, phenomenology, content analysis<br>In the methods section I explain that this is an ethnographic study using observation and interviews and that data analysis followed an ethnographic approach through the lenses of mindlines and self-management.	5
Participant selection<br>10. Sampling How were participants selected? e.g. purposive, convenience, consecutive, snowball<br>I collected observational data during 250 hours in the practice. I selected interview participants using maximum variation purposive sampling to ensure a mix of profession, gender and years in practice.	6
11. Method of approach How were participants approached? e.g. face-to-face, telephone, mail, email<br>Observation participants were recruited at the beginning of each encounter with the practice. I recruited interview participants from the practice and local pharmacy.	6
12. Sample size How many participants were in the study?<br>Observation involved many participants and 16 interviews were completed	6
13. Non-participation How many people refused to participate or dropped out? Reasons?<br>On occasion I exited consultations at the request of the patient, practitioner or of my own volition<br>A few practitioners declined to take part in interviews due to time constraints.	5
Setting<br>14. Setting of data collection Where was the data collected? e.g. home, clinic, workplace<br>Observational data was collected in a GP practice. Interviews were conducted in their workplace.	6
15. Presence of non-participants Was anyone else present besides the participants and researchers?	6

For peer review only

BMJ Open Practitioner eczema mindlines COREQ 4.7.18

Observational data involved numerous individuals. Interviews were conducted individually.	
16. Description of sample What are the important characteristics of the sample? e.g. demographic data, date<br>Interview participants were sampled by profession, gender and years in practice.	Table 2
Data collection<br>17. Interview guide Were questions, prompts, guides provided by the authors? Was it pilot tested?<br>A topic guide was used for interviews	Table 1
18. Repeat interviews Were repeat interviews carried out? If yes, how many?<br>No repeat interviews were carried out	NA
19. Audio/visual recording Did the research use audio or visual recording to collect the data?<br>Interviews were audio recorded and observational data recorded in field notes	6
20. Field notes Were field notes made during and/or after the interview or focus group?	6
21. Duration What was the duration of the interviews or focus group?<br>Interviews lasted from 22-40 minutes	6
22. Data saturation Was data saturation discussed?<br>Data sufficiency was achieved when no new sources of knowledge were identified in interviews.	8
23. Transcripts returned Were transcripts returned to participants for comment and/or correction?<br>No	NA
**Domain 3: analysis and findings**<br>*Data analysis*<br>24. Number of data coders How many data coders coded the data?<br>I analysed the data independently and subsequently discussed with research, clinical and lay colleagues who corroborated initial interpretations.	8
25. Description of the coding tree Did authors provide a description of the coding tree?<br>No	NA
26. Derivation of themes Were themes identified in advance or derived from the data?<br>Themes were derived inductively from the data	8
27. Software What software, if applicable, was used to manage the data?<br>No	NA
28. Participant checking Did participants provide feedback on the findings?<br>No	NA
Reporting<br>29. Quotations presented Were participant quotations presented to illustrate the themes / findings? Was each quotation identified? e.g. participant number<br>Quotations are provided and profession of participant is identified.	9 - 14
30. Data and findings consistent Was there consistency between the data presented and the findings?<br>Yes.	9 - 14
31. Clarity of major themes Were major themes clearly presented in the findings?<br>Yes.	9 - 14
32. Clarity of minor themes Is there a description of diverse cases or discussion of minor themes?<br>Yes, I present a spectrum of practitioners and variations of mindlines.	9 - 14

For peer review only

BMJ Open

Knowledge mobilisation: An ethnographic study of the influence of practitioner mindlines on atopic eczema self-management in primary care in the United Kingdom

Journal:	*BMJ Open*
Manuscript ID	bmjopen-2018-025220.R2
Article Type:	Research
Date Submitted by the Author:	24-May-2019
Complete List of Authors:	Cowdell, Fiona; Birmingham City University, Faculty of Health Education and Life Sciences
**Primary Subject Heading**:	Dermatology
Secondary Subject Heading:	Evidence based practice, General practice / Family practice, Health services research, Qualitative research
Keywords:	Ethnography, Knowledge mobilisation, Mindlines, PRIMARY CARE, Self-management, Atopic eczema

SCHOLARONE™
Manuscripts

Marked copy BMJ Open KM Practitioner mindlines R2 24.5.19

Article title: Knowledge mobilisation: An ethnographic study of the influence of practitioner mindlines on atopic eczema self-management in primary care in the United Kingdom

Author information:
Fiona Cowdell
DProf, RN
Professor of Nursing and Health Research
Faculty of Health, Education and Life Sciences
Birmingham City University
220 Ravensbury House
Westbourne Road
Edgbaston
Birmingham
B15 3TN
UK
Telephone: +44 (0)121 300 4345
Email: Fiona.cowdell@bcu.ac.uk
orcid.org/0000-0002-9355-8059

Word count: 5324

Number of figures and tables: one figure and four tables

Keywords: atopic eczema, ethnography, knowledge mobilisation, mindlines, primary care, self-management

For peer review only

For peer review only - http://bmjopen.bmj.com/site/about/guidelines.xhtml

Marked copy BMJ Open KM Practitioner mindlines R2 24.5.19

Knowledge mobilisation: an ethnographic study of the influence of practitioner mindlines on atopic eczema self-management in primary care in the United Kingdom

Abstract

Objective: To explore how atopic eczema specific mindlines are developed by primary care practitioners.

Design: Ethnographic study.

Setting: One large, urban general practice in central England.

Participants: In observation, all practitioners and support staff in the practice and in interviews a diverse group of practitioners (n=16).

Results: Observation of over 250 hours and interview data were combined and analysed using an ethnographic approach through the lenses of mindlines and self-management. Three themes were identified: beliefs about eczema, eczema knowledge and approaches to self-management. Eczema mindlines are set against a backdrop of it being a low priority and not managed as a long-term condition. Practitioners believed that eczema is a simple to manage with little change in treatments available and prescribing limited by local formularies. Practice is largely based on tacit knowledge and experience. Self-management is expected but not often explicitly facilitated. Clinical decisions are made from knowledge accumulated over time. Societal and technological developments have altered the way in which practitioner mindlines are developed; in eczema, for most, they are relatively static.

Conclusions: The outstanding challenge is to find novel, profession and context-specific, simple, pragmatic strategies to revise or modify practitioner mindlines by adding reliable and useful knowledge and by erasing outdated or inaccurate information thus potentially improve quality of eczema care.

Strengths and limitations

- First ethnographic study to examine the development of atopic eczema specific mindlines
- Diverse sample primary care practitioners
- Ethnographer was a lone researcher
- Results may be context specific

For peer review only - http://bmjopen.bmj.com/site/about/guidelines.xhtml

For peer review only

Marked copy BMJ Open KM Practitioner mindlines R2 24.5.19

Introduction

Atopic eczema (hereafter "eczema") is a common, long-term skin condition affecting around one in five children and one in twelve adults in the United Kingdom (UK). It can have a detrimental impact on wellbeing and quality of life and globally is one of the fifty most burdensome diseases (1). Eczema is mainly treated in primary care (2). People may seek advice from general practitioners (GPs), practice nurses (PNs), nurse practitioners (NPs), health visitors (HVs), community pharmacists (CP) and pharmacy counter staff (PCS).

GP consultations are often unsatisfactory for both patient (3) and practitioner (4, 5) with GPs dominating encounters and using avoidance tactics (6) and there being significant dissonance between patient / parent and GP beliefs about assessment and treatment (6). Many GPs have limited specialist dermatology knowledge (7). Nurse consultations, albeit in secondary care, tend to be more positively evaluated (8, 9) and minimal research has been conducted into the contribution of HVs. Research into the role of the CP in dermatology care is limited (10) and expertise may be suboptimal (11) despite CPs reports of being at least reasonably confident in their role (12). The role of the pharmacy counter assistant is equally under-researched although they are often first point of contact for customers and may offer health advice independent of pharmacists (10, 13).

The mainstay of eczema treatment is the regular application of emollients, at least daily and often for many years, with or without intermittent topical steroids and calcineurin inhibitors. Non-adherence results from the high self-management demand of applying topical treatments (14) but also lack of information and conflicting advice from different health professionals (15). Despite available evidence (for example the Global Resource for Eczema Trials database (16) and the National Institute for Health and Care Excellence, Guideline for Eczema (17)) providing evidence-based treatment appears to be a challenge for health professionals managing eczema (18).

For peer review only - http://bmjopen.bmj.com/site/about/guidelines.xhtml

Self-management is a policy imperative which can improve disease outcomes and quality of life for people living with long-term conditions (19). Strategies to support eczema self-management are poorly understood, have limited availability, can be costly and have variable impact (20). Eczema is not classified as a long-term condition in the same way as other illnesses for example asthma (21).

Primary care practitioners are expected to deliver evidence-based practice (EBP). Evidence based medicine (EBM) was originally the preserve of doctors and was defined as “the conscientious, explicit, and judicious use of current best evidence in making decisions about the care of individual patients” (22). Over time, other professions have embraced EBP but this has, at times, been conceptualised as a set of research-based facts which if disseminated to practitioners will ensure more standardised, high quality care (23); this notion is now largely dismissed (24). Primary care practitioners face particular challenges in EBP given the volume of information they need and information overload is a real problem (25).

The study of knowledge mobilisation (KM) is growing exponentially in health care, at its simplest it is “moving knowledge to where it can be most useful” (26). KM involves determined efforts to create, share and use research and other forms of knowledge predicated on the understanding that to be effective KM activity must be relational, constructed from social interaction and context-specific (27-29).

Mindlines, developed from a primary care based ethnographic study (30) offer a “real world” approach to mobilising knowledge and changing clinical practice. Mindlines are “collectively reinforced, internalised tacit guidelines” which underpin clinical decision-making (30). They build on the work of Polyani (31) and Nonaka and Takeuchi (32) who propose that knowledge is not necessarily conscious and explicit, and that tacit knowledge in the form of unconscious schemata and technical know-how, are dominant influencers of action compared with formal codified knowledge. Gabbay and LeMay (28) suggest that mindlines are based on flexible, embodied and intersubjective understanding of knowledge that is grounded in the acceptance that there are multiple realties and that knowledge is context-specific. Mindlines represent a complex amalgamation of knowledge gathered from many sources for example, communication with colleagues and

For peer review only - http://bmjopen.bmj.com/site/about/guidelines.xhtml

Marked copy BMJ Open KM Practitioner mindlines R2 24.5.19

opinion leaders in the field and from tacit knowledge developed over time (28). In their original work, Gabbay and LeMay (30) examined the construction of mindlines across primary care. A subsequent synthesis of 10 years of mindline literature (n=340) reports that they have been conceptualised and used in four distinct ways. “Nominal” in which the term was used in name only, sometimes with a degree of scepticism, “in practice” examining how mindlines are developed and spread in everyday practice, “theoretical and philosophical” in which the aim was to extend existing theory and “solution focused”, exploring ways in which mindlines can be influenced. Solution focused papers (n=28) emphasise the importance of collaborative learning, relationship building and effective leadership in the development of valid, collective, evidence-based mindlines. This review reveals a paucity of information about development or strategies to amend condition specific mindlines (33). Repeating the search strategy utilised for this review in 2018 revealed an abundance of further related literature but little directly addressing condition specific mindlines or how they may best be amended.

Given the prevalence of eczema, the challenges of primary care consultations and the high self-management demand, it is prudent to investigate the way in which eczema mindlines are constructed by practitioners. This will inform understanding of mindlines “in practice” and will underpin future “solution focused” work to develop novel, context-specific, simple and pragmatic strategies to revise or modify eczema mindlines by adding reliable and useful knowledge and by erasing outdated or inaccurate information, thus potentially improve quality of eczema care and self-management.

Method

Aim

To understand construction of healthcare practitioner atopic eczema mindlines in primary care.

Design

An ethnographic approach was employed. Ethnography is founded in anthropology and is concerned with the systematic study of people and cultures (34). Data is collected through extensive observation with

For peer review only - http://bmjopen.bmj.com/site/about/guidelines.xhtml

informal conversations, field notes and interviews (35, 36). Data was collected in one large general practice in England.

Setting, participants and process

Data were collected by the author, a nurse and researcher, from January - June 2017. The General Practice was identified by a local Clinical Research Network. It was a research and education active urban general practice in a demographically diverse and deprived area of England with a patient population of approximately 10,000. Observations were also conducted in a community pharmacy adjacent to the practice, which was used by most patients. No practitioners reported a special interest in dermatology. In preparation for data collection the researcher attended two practice meetings to outline conduct of the study. Data were collected in more than 250 hours of observation during all surgery opening hours. The role of social-participant-as-observer, that is, predominantly observer with some social functions such as cleaning couches was taken (37). Observation began with the reception team to understand the day-to-day working of the practice. Observation of consultations with GPs, GP trainees and locums, nurses, health visitors in baby clinics, held on the practice premises, and pharmacy staff followed. GP telephone consultations were listened to and discussed with the practitioner. Field notes were documented and informal conversations either written contemporaneously or audio-recorded. Entire clinics were attended regardless of presenting complaint, to gain understanding in the context of other long-term conditions. Between consultations practitioners recounted recent eczema consultations. Available documentation was reviewed. Single, semi-structured interviews using a topic guide (Table 1) were conducted with practitioners from each profession (n=16) (Table 2) using maximum variation purposive sampling (38) to ensure a mix of job role and level of experience. A predominance of female participants was reflective of the profile of the healthcare team. The complete dataset is summarised in Table 3.

Table 1: Practitioner interview topic guide

- Do you have any special interest in skin health?
- How much contact do you have with patients with eczema?

For peer review only - http://bmjopen.bmj.com/site/about/guidelines.xhtml

For peer review only

Marked copy BMJ Open KM Practitioner mindlines R2 24.5.19

- What sort of treatments do you use most often?
- How do you decide on a particular treatment?
  - What impact does the local formulary have on your prescribing?
- How much are you able to advise patients on how to care for their eczema?
  - Concordance etc
- How do you update your own knowledge about eczema?
- How could we best get research information to use in your practice?
  - What methods do you use now?
  - Can you give any specific examples?
- Do patients come with their own ideas about the treatment they need?
- How much do you and your patient share the decision about what treatment to use?
- How do you reconcile patient's needs with what is available?
- Do you refer patients to any external sources of information?

Table 2: Demographic details of interview participants

**Role**	**Gender**	**Years in current role**
Health visitor	Female	10
GP	Male	35
GP Trainee	Female	2
Practice nurse	Female	31
Practice nurse	Female	32
Pharmacist	Male	8
GP Trainee	Female	5
Pharmacist	Female	12
Pharmacy counter staff	Female	10
Pharmacy counter staff	Female	17

For peer review only - http://bmjopen.bmj.com/site/about/guidelines.xhtml

GP Trainee	Female	7
GP	Female	6
GP	Female	5
Health visitor	Female	2
Health visitor	Female	2
Health visitor	Female	3

Table 3: Complete dataset

**Observations and informal interviews**
1 General practice
10 sessions observing reception and waiting room
9 sessions observing in baby clinics
2 sessions observing in community pharmacy
24 sessions observing GPs
5 sessions with practice manager
Multiple informal meetings and one to one informal discussions
4 practice meetings
6 debriefs with GP trainees
**Formal interviews**
16, details provided in table 2
**Documentary sources**
Local prescribing guidelines
Online guidance accessed by practitioners during observation

Interviews were conducted in the workplace and lasted from 22-40 minutes. Data sufficiency was achieved when no new insights were forthcoming (39). For completeness documents and websites were reviewed

For peer review only - http://bmjopen.bmj.com/site/about/guidelines.xhtml

For peer review only

Marked copy BMJ Open KM Practitioner mindlines R2 24.5.19

including the NICE Clinical Guidance for Eczema (17), the local emollient formulary and the Clinical Knowledge Summary (40) and GP notebook pages (41) for eczema.

Data collection and analysis were iterative with initial findings being used to guide further collection (42). Audio-data were professionally transcribed and transcripts read against the recording by the researcher to confirm accuracy. Data analysis was completed independently by the researcher, though the lenses of mindlines and self-management. Transcripts and field notes were read in full to get a sense of the data as a whole, and then manually coded, categorised and merged into themes and annotated with researcher inductive interpretations (see table 4 for worked example). Post theme development, relevant sections of the data were revisited to ensure authentic interpretation and use of participant language.

Table 4: Example of data analysis process

Codes (from interview and observational data)	Categories	Theme
GP interview<br>• Eczema "simple to treat" nothing much has changed over the years – it's bread and butter to us	It's simple to treat	Beliefs about eczema
HV interview<br>• Basics are the same, but there's lots of personal preference		
GP interview<br>• Common complaint "know by heart"		
Observational data<br>• Perception from GPs that it's a straightforward condition, treatment is fairly standard and that there is limited need for further knowledge. Intranet rarely used but fairly standard set of resources for GPs		
GP interview<br>• Software will fire up a message if another product should be used	No need to think too much	
Pharmacist interview<br>• Script Switch – computer tells you if you are prescribing the wrong thing and suggests an alternative		
Observational data<br>• Belief that guidance is more about cost that research		
Observational data<br>• Eczema is not a condition that is mentioned in "learning" interactions such as debriefs		

Reflexivity

Reflexivity was maintained throughout the study with particular attention being paid to subjectivity and positioning as a nurse and skin health researcher; pre-understandings were consciously set aside (43).

For peer review only - http://bmjopen.bmj.com/site/about/guidelines.xhtml

Marked copy BMJ Open KM Practitioner mindlines R2 24.5.19

Patient and Public Involvement

Lay people, from an eczema support group, were involved in the development of the research question and in planning the design of the study. They contributed through one meeting and a series of email exchanges.

Results

Data analysis resulted in three themes: beliefs about eczema; eczema knowledge and approaches to self-management. Each is discussed with examples from the data below.

For peer review only

Theme 1: Beliefs about eczema

Eczema was consistently viewed as a *"bread and butter"* [GP] condition that accounted for many consultations. However, although 19.5% of the practice population was recorded as having some type of eczema few consultations primarily for this condition were observed. Analysis of patient reported reason for GP consultation for a typical week during observation revealed that 26/627 (4.1%) of reasons were skin related with none citing eczema as the primary complaint. No observed face-to-face consultations were primarily for eczema; it was reported as a secondary concern in a small number of number of GP consultations and more often to HVs in baby clinics. This resulted in eczema necessarily being given limited attention *"it's often a secondary problem and there's only time to deal with one problem per consultation"* [GP]. Telephone consultations with GPs were witnessed and patients were observed to consult with pharmacy staff about their eczema. Practitioners mainly viewed eczema as a nuisance condition requiring limited knowledge to treat effectively, *"eczema is simple to treat, nothing much has changed over the years"* [GP] and *"the recipe doesn't change* [GP]".

Some GPs described eczema as a *"catch up"* [GP] consultation when clinics were over-running. GPs and nurses noted the absence of specific external incentives for long-term eczema management and that it was a condition without the *"red flags"* [GP] which trigger treatment escalation or referral. They described treatment options as straightforward involving emollients with or without intermittent topical steroids.

For peer review only - http://bmjopen.bmj.com/site/about/guidelines.xhtml

Few mentioned calcineurin inhibitors or other available medications. Most practitioners considered emollients to be a homogenous group of preparations all with similar properties, although a few differentiated in terms of viscosity and texture. Pharmacy staff and HVs were familiar with a broader range of emollient products and were more likely to offer suggestions for over the counter preparations. This was in part because no HVs in this study were able to prescribe. GPs were reluctant to prescribe topical steroids or other treatments unless absolutely necessary. PNs rarely saw patients with atopic eczema.

Practitioners recognised that eczema could have a negative impact on wellbeing and quality of life but this was not often reflected in the care offered. Treatment was mainly in reaction to a flare rather than there being a long-term plan of care. Generally patients were able to access regular repeat prescriptions for emollients and practitioners expressed a level of frustration when they presented with a flare having not requested or used the prescribed treatments. Although 'safety netting' was always in place, planned follow-up consultations were not suggested. Empathy for patients was most evident in practitioners who had personal experience of eczema, they articulated a varying level of understanding about the differences between products, regardless of available empirical evidence, and the extent to which personal preference influenced concordance. Pharmacy counter staff were the most conversant with the differences between emollient products having tried samples, and they were most likely to share this knowledge with patients / customers verbally and in leaflets.

Although eczema was viewed as a frequent reason for consultation, it was mainly presented as a secondary concern and so dealt with swiftly. Eczema was considered simple to treat with little change over time although practitioners with personal experience of eczema were more aware of the challenges of self-management and tolerant of personal treatment preferences.

Theme 2: Atopic eczema knowledge

Beliefs about eczema influenced the formation of mindlines and for most mindlines were set against a backdrop of eczema being a low priority condition and a perception of unchanging treatment options which

For peer review only - http://bmjopen.bmj.com/site/about/guidelines.xhtml

For peer review only

were constrained by local prescribing guidelines (Figure 1). Many practitioners described AE as a common conditions for which you "*know [treatment] by heart* [GP]" and likened his response to using a "*satnav …………you stop thinking, the little NHS boxes [on the computer] tell you what to prescribe* [GP]".

Most practitioners reported that their eczema knowledge was based on their initial education and recognised "*pre-reg derm education was very, very basic"* [GP]. A few had completed dermatology placements during GP training but reported seeing little eczema. One experienced GP recounted learning from a consultant, her practice was unchanged as she had "*learnt from a consultant many years ago and never heard anything to contradict it"* [GP]. PNs and GPs were aware of available dermatology education but did not attend as it was a low priority and costly, "*there is training but you have to pay"* [PN] and they preferred to "*avoid reps and sponsored sessions"* [GP]. HVs reported that skin health was never an educational priority. Pharmacist's knowledge was updated through e-bulletins from different sources and covered only changes in, and availability of, medications. Only PCS received eczema specific education by attending regular seasonal sessions provided by their employer. Although deemed to be useful, particularly as they tried products and were advised on correct application, the educational experience was sometimes suboptimal as one reported how she was "*shamed into remembering"* [PCS] session content.

Local emollient guidelines underpinned many prescribing decisions so practitioners did not need to think as "*software will fire up a message if another product should be used"* [CP]. Changes to guidelines were ascribed to cost and "*what was in vogue"* [GP]. Practitioners were not concerned about these changes stating for example, "*aqueous cream, they've gone off that idea for some reason"* [PCS] and "*Zero products are the ones that are currently on trend"* [GP trainee]. Whilst some prescribers stuck rigidly to prescribing the cheapest product, "*I try to be good and prescribe the cheaper side of things"* [GP trainee], others were more flexible according to their own or the patient's preference. However, deviations from the formulary were rare on the basis that "*local formulary is very constraining and you'd have to be able to justify why you'd prescribed anything else"* [GP]. Exceptions were observed in the baby clinic and in pharmacy practice where patients were often informed about a wider range of emollients that could be purchased over the

For peer review only - http://bmjopen.bmj.com/site/about/guidelines.xhtml

counter. For those who paid a prescription charge this could often be more cost effective. PCS suggested that they were able to advise patients readily as they had "*tried samples so you can tell the customers what they feel like*" [PCS].

Other knowledge sources contributed to eczema mindlines. All staff, with the exception of experienced GPs, used internet searches most commonly the online resources GP Notebook and Clinical Knowledge Summaries. Useful websites were often book-marked and visited in preparation for a consultation rather than alongside the patient. If information was not located almost immediately the practitioner switched to another website "*we're hard wired for speed now*" [GP] and "*dipped into what's relevant*" [GP] as and when required. None mentioned existing NICE Eczema Guidance. Local emollient guidelines existed and influenced the prescribing practice of most practitioners, however others were unaware of these and some found them hard to access. GPs and HVs used different emollient guidelines and this caused confusion for patients when they consulted both. A member of pharmacy staff noted the need for "*a synchronised approach so patients don't get confused*" [PCS]. Practitioners also experienced confusion when offering advice on treatment application, for example "*treatment is a bit arbitrary – for example should you advise steroid or emollient first?*" [HV].

Practitioners learnt from each other to a limited extent, most often within their professional groups. They recognised "*we learn both good and bad habits from each other*" [PN]. Opportunities for shared learning had reduced as there was little time to meet up and in-house teaching for GPs and PNs had "*fallen by the wayside*" [GP] due to staff sickness and pressure of work. One GP reported "*phoning a friend*", now a consultant dermatologist, when she needed advice. GPs reported learning from trainees during debrief sessions but could not recall ever having discussed eczema. Trainees exhausted all available information sources before seeking advice from a GP. HVs and PNs met more frequently and exchanged knowledge more regularly, although eczema was not a condition of interest.

For peer review only - http://bmjopen.bmj.com/site/about/guidelines.xhtml

Practitioners expressed varied views on the value of patient knowledge and experience and the extent to which it influenced care. PNs, HVs and pharmacy staff respectively reported that they routinely "*ask patient what they have tried already*" [PN], "*see what's worked for them*" [HV] and "*listen and learn from customers*" [PCS] and used this information as a basis for treatment advice. Others listened to patients with a degree of scepticism but acquiesced to patient preference, "*patients often have fixed ideas [about emollients] and I try to accommodate these*" [GP]. A few were less receptive, for example "*I try to use guidelines and the formulary ………… patient experience stuff can be counterproductive*" [GP trainee] and others suggested that their wider experience overrode the patients personal preferences and experiences "*experience wise I've found a lot of people get on with it [particular emollient]*" [GP] and therefore that was what would be prescribed.

Only the most experienced practitioners spontaneously articulated the existence of tacit knowledge stating, "*it's a perpetual exercise … adding on knowledge and skills*" [GP] and "*built up knowledge over time*" [PN]. Others pointed to more concrete sources of knowledge. All practitioners understood reliability of evidence to a greater or lesser extent.

Eczema knowledge was constructed from different sources by individual professions. Nursing and medical staff perceived a limited need to update their knowledge as eczema care was viewed as having changed little over time. Exceptions to this were practitioners who had personal experience of eczema and pharmacy staff who regularly updated their mindlines using informal and formal sources of knowledge.

Theme 3: Approaches to self-management

In principle, all practitioners supported self-management of eczema but recognised the difficulties of achieving this in practice particularly without formal recognition as a long-term condition (LTC). Some practitioners routinely used techniques to support self-management for patients with other LTCs. Strategies included for example, "*finding out patients' expectations*" [PN], "*tailoring knowledge to the person*" [GP], "*start with what the patient understands and then fill in the gaps*" [GP], "*give patients a map*

For peer review only - http://bmjopen.bmj.com/site/about/guidelines.xhtml

Marked copy BMJ Open KM Practitioner mindlines R2 24.5.19

of management" [GP], "*instil confidence"* [GP] and "*reinforce that self-management is good"* [GP trainee]. A few GPs used specific techniques such as "*short bursts of CBT"* [GP], "*motivational interviewing techniques ……. compressed to fit in consultation"* [GP] and "*behaviour modification ………… not a one consultation job"* [GP]. Even practitioners who did not articulate using strategies to support self-management integrated them in practice for many LTCs. However they were rarely observed or discussed in relation to eczema.

Most eczema care was reactive when patients presented with a flare and talk of eczema care was almost exclusively about treatment options. Virtually no attention paid to ensuring that the patient understood the condition and actions they could take to avoid the relentless cycle of flares. The most tangible contribution to self-management was the availability of repeat prescriptions for emollients but advice to use these consistently was lacking. Barriers to self-management were observed, for example the appointment system often precluded patients seeing the same GP over time, so treatment could be altered without the benefit of fully understanding the patient journey to date. Contradictory advice given by practitioners and a lack of faith in patient's ability to judge when they needed to use topical steroids and to use them safely presented significant barriers to successful self-management. Practitioners suggested they "*need to see patients before prescribing [topical] steroids"* [GP]. One GP stated that "*sensible*" patients may get steroids on repeat but struggled to define sensible in this context. Pharmacy staff did not recognise their contribution to self-management per se, but recognised the positive impact they had on eczema management through "*actually taking notice of what they're telling me"* [PCS] and perceived "*they do trust me ………………… I'm well known in the local community"* [PCS] and were therefore easy for customers to speak with.

Whilst recognising the need for self-management the fact that eczema is not categorised as a long-term condition limited how much patients were supported to self-manage and at times healthcare systems could hinder attempts.

For peer review only - http://bmjopen.bmj.com/site/about/guidelines.xhtml

Discussion

This study offers new insights into how primary care practitioners construct atopic eczema specific mindlines. Practitioner mindlines are predominantly set against a back drop of eczema being a low priority, due to a combination of not being viewed as an LTC and so lacking external incentives, and the perception of available treatments being standard use of emollients and topical steroids, which changes little over time and is constrained by prescribing guidelines. This led to an assumption that there was little need to amend mindlines. Eczema mindlines were developed early in their career by many practitioners and were relatively static amongst GPs, PNs and HVs, except for those with direct personal experience of eczema. Mindlines of pharmacy staff were regularly modified through a combination of education provided by their employer, electronic updates from professional bodies and interactions with customers. The latter was particularly influential for the PCSs as they generally had more time to listen and had built up trusting relationships with the customers over time.

This study is one of few to apply mindline theory to a specific condition across a broad range of practitioners. In particular it identifies important differences in the way in which eczema mindlines are developed and so may best be amended for individual practitioner groups. This study conforms with conventions of robust qualitative work in that it is rigorous (coherent and sufficiently well reported to be open to external audit), relevant (enriches understanding of the subject), resonant (resonates with readers experiences and understandings) and reflexive (subjectivity of the author is acknowledged) (44). Limitations include the ethnographer being a lone worker and data analysis being completed by the researcher alone, however this is mitigated by conversations with participants to check understandings. As data were collected in one general practice, findings may not be transferable but the diversity of participants should minimise this risk (45). Additionally no nurse practitioners were included as, at the time of data collection, none were employed in the practice.

As with the original conceptualisation of Gabbay and leMay (30), practitioner eczema mindlines are composed over time, from a range of evidence sources which rarely embrace direct use of research.

For peer review only - http://bmjopen.bmj.com/site/about/guidelines.xhtml

Marked copy BMJ Open KM Practitioner mindlines R2 24.5.19

Gabbay and le May (28) point to the critical nature of knowledge-in-practice-in-context in which in each context new knowledge is converted by the complex social processes of the Socialization, Externalization, Combination, Internalization spiral (32). Context was central in the formation of eczema mindlines but was informed more by long-held beliefs and national policy than by local context. Key differences in this study are that mindline development has evolved alongside the changing nature of primary care where practitioners, particularly GPs, appear to work more in isolation than as part of a community with "coffee room chat" (46) appearing much reduced. In parallel, available online resources have spiralled thus potentially reducing the need to confer with others. This challenges the notion that mindlines are heavily reliant on professional interactions (28). The static nature of eczema mindlines and the beliefs underpinning eczema care meant that they were accessed using fast, automatic, System 1 thinking rather than the more deliberative, conscious, slow and effortful System 2 approach (47).

Few studies have investigated condition specific mindlines with the exception of a Tanzanian study of malaria diagnosis (48), however the depiction here is more akin to rules of thumb or heuristics. A comprehensive commentary on mindlines identifies 76 papers categorised as "in practice", that is studies of how mindlines are developed, many of these used the term to mean consulting with colleagues (33). A smaller number were faithful to the original Gabbay and Le May's conceptualisation but add little by way of new understanding. More recently, Wieringa and colleagues (49) investigated mindlines development in online clinical communities concluding that they offered collective, dynamic settings and suggest implicitly that they may be areas for mindline amendment. Whilst online communities may appeal to some practitioners, this will not be so for all.

In this study eczema was consider low priority. These beliefs are longstanding with surveys suggesting that both patients and practitioners perceive dermatology as a poor relation in healthcare (50-52) and Magin and colleagues (4) describing 'dismissive' and 'unsympathetic' attitudes amongst GPs. Eczema appears to be considered as "health problem which is not an illness" (53) and therefore less legitimate and worthy than other conditions. Ambivalence about eczema specific learning was in contrast to a survey which

indicated a desire for new knowledge, particularly in the form of education delivered by consultants (54); inevitably GPs completing the survey would be those with an interest in dermatology. The dermatology community has used many strategies to make research findings accessible to all with limited success (55). In contrast with this study in which treatment for eczema was viewed as simple others report GPs uncertainty about managing eczema (56).

Achieving change in primary care practice is challenging, interventions most likely to influence practice demonstrate evidence of benefit, are simple to use and adaptable to local context (57). The context of eczema mindlines, that it is a low priority condition with a limited repertoire of treatment options, is unlikely to change in the foreseeable future. If, like other LTCs, eczema was recognised in the Quality and Outcomes Framework (58) patients may benefit from the accelerated trends towards systematic management (59). Practitioners in primary care are expert generalists (60) and are expected to have knowledge of many conditions for which there is wealth of available evidence. This may lead to information overload for which coping strategies are needed. Bate and colleagues (61) describe “satisficing” that is, curtailing the amount of information gathered to enable them to make a “good enough” decision.

In many ways it can be argued that treatment of eczema in primary care is relatively straightforward and that amendment of mindlines to adjust thinking about emollients and removal of outdated information about topical steroid use could make a significant change in practice that would improve both patient experience and self-management practices. Brevity and accessibility of information is key as practitioners have been found to judge the usefulness of new knowledge as function of its relevance x validity ÷ by the work needed to access it (62). It is possible that straightforward messages could be conveyed through media such as aphorisms, “succinct sayings that offer advice” (63) or actionable nuggets “knowledge translation tools designed to provide …….. concise practical information about the most prevalent and pressing primary care needs of patients” (64). This approach offers the opportunity to compensate for the loss of professional wisdom through personal communication by transmitting concentrated wisdom and guidance in a different way (63).

For peer review only - http://bmjopen.bmj.com/site/about/guidelines.xhtml

Efforts to amend GPs, PNs and HVs mindlines need to be accessible via rapid System 1 thinking. Interventions should be specific, practical, tailored, relevant and rapidly delivered information which can readily be assimilated, or as participants in this study described it, a "no faff" approach. Given their time constraints and information gathering habits, any new information would best be delivered individually rather than in a group setting and available online and possibly in other formats.

The role of the community pharmacist in eczema care is evolving partly in response to Pharmaceutical Services Negotiating Committee guidance on Medicines Use Reviews (65), New Medicine Service (66) and Minor Ailment Service (67). Forthcoming changes in availability of emollients on prescription may increase their role further. Pharmacy staff described eczema mindline development as a more collective experience than other practitioners and valued learning from each other and from customers. They may be open to group approaches to update and remove redundant information from their mindlines and this would need to be brokered through both professional and employing organisations.

Conclusion

This ethnographic study provides new understandings about the development of atopic eczema specific mindlines in different practitioner groups in primary care. The outstanding challenge is to find novel, context-specific, simple, pragmatic strategies to revise or modify these mindlines by adding reliable and useful knowledge and by erasing outdated or inaccurate information using strategies that are most appropriate to each profession. Mindline amendment has the potential to improve self-management and quality of eczema care through the delivery of consistent, evidence-based care.

For peer review only - http://bmjopen.bmj.com/site/about/guidelines.xhtml

Marked copy BMJ Open KM Practitioner mindlines R2 24.5.19

Contribution statement

FC is the sole contributor to this paper

Funding

Fiona Cowdell is funded by a National Institute for Health Research, Knowledge Mobilisation Research Fellowship, KMRF-2015-04-004.

Disclaimer

This paper presents independent research funded by the National Institute for Health Research. The views expressed are those of the author and not necessarily those of the NHS, the NIHR or the Department of Health and Social Care.

Ethical approval

The study was approved by a National Health Service REC (16/YH/0252). Process consent was used for observation, on each occasion informal conversations were used to re-check participant's willingness to be observed. Patients were informed about the study by practitioners and when necessary the researcher exited individual consultations, either at the request of the patient, the practitioner or using personal judgment, although this was infrequently needed. Written consent was taken for audio-recorded interviews. Interview participants consented to publication of anonymised information.

Competing interests

None to declare

Acknowledgements

Thanks to Amanda Roberts who have given invaluable lay feedback on the planning and design of this study. Thanks to James Mycock, Birmingham City University for the mindline illustration and to Professors Hywel William and Stephen Timmons, University of Nottingham for their valuable feedback on earlier iterations of this manuscript.

Data sharing statement

The datasets generated and/or analysed during the current study are not publicly available as they are not designed to be re-analysed by others but are available from the corresponding author on reasonable request.

For peer review only - http://bmjopen.bmj.com/site/about/guidelines.xhtml

Marked copy BMJ Open KM Practitioner mindlines R2 24.5.19

References

1. Hay RJ, Johns NE, Williams HC, et al. The global burden of skin disease in 2010: an analysis of the prevalence and impact of skin conditions. J Invest Dermatol. 2014; 134: 1527-1534.
2. Schofield J, Grindlay D, Williams H, Skin conditions in the UK: a health care needs assessment. Centre of Evidence Based Dermatology, University of Nottingham; 2009.
3. Cowdell F. Knowledge mobilisation: an ethnographic study of the influence of lay mindlines on eczema self-management in primary care in the United Kingdom. BMJ Open. In press
4. Magin P, Adams J, Heading G, Pond C. Patients with skin disease and their relationships with their doctors: a qualitative study of patients with acne, psoriasis and eczema. Med J Aust. 2009; 190:62-64
5. Santer M, Burgess H, Yardley L, et al. Experiences of carers managing childhood eczema and their views on its treatment: A qualitative study. Br J Gen Pract. 2012; 62:261-267.
6. Powell K, Le Roux E, Banks J, et al. GP and parent dissonance about the assessment and treatment of childhood eczema in primary care: a qualitative study. BMJ Open. 2018; 8:e019633.
7. Rübsam M, Esch M, Baum E, et al. Diagnosing skin disease in primary care: A qualitative study of GPs' approaches. Fam Pract. 2015; 32: 591-595.
8. Nicol NH, Ersser SJ. The role of the nurse educator in managing atopic dermatitis. Immunol Allergy Clin. 2010; 30:369-383.
9. Rolinck-Werninghaus C, Trentmann M, Reich A, et al. Improved management of childhood atopic dermatitis after individually tailored nurse consultations: a pilot study. Pediat Allergy Immunol. 2015; 26: 805-810.
10. Tucker R, Stewart D. Why people seek advice from community pharmacies about skin problems. Int J Pharm Pract. 2015; 23(2): 150-153.
11. Tucker RP, MacLure K, Paudyal V, et al. An exploratory study of community pharmacist diagnosis and management of dermatitis and acne. Selfcare J. 2017; 8(2):1-10
12. Tucker R. The medicines use review in patients with chronic skin diseases: are pharmacists doing them and how confident are they? Int J Pharm Pract. 2013; 21:202-204.
13. Banks J, Shaw A, Weiss MC. The community pharmacy and discursive complexity: a qualitative study of interaction between counter assistants and customers. Health Soc Care Community. 2007; 15:313-321.
14. Santer M, Burgess H, Yardley L, et al. Managing childhood eczema: Qualitative study exploring carers' experiences of barriers and facilitators to treatment adherence. J Adv Nurs. *2013*; 69:2493-2501.
15. Teasdale EJ, Muller I, Santer M. 2017. Carers' views of topical corticosteroid use in childhood eczema: a qualitative study of online discussion forums. Br J Dermatol. 2017; 176(6):1500-1507.
16. Global Resource for Eczema Trials (GREAT) database http://www.greatdatabase.org.uk/GD4/Home/Index.php Accessed 4.7.18
17. National Institute for Health and Care Excellence. Atopic eczema in under 12s: diagnosis and management. Clinical guideline [CG57] 2007 https://www.nice.org.uk/Guidance/CG57. Accessed 4.7.18
18. Powell K, Le Roux E, Banks JP, et al. Developing a written action plan for children with eczema: A qualitative study. Brit J Gen Pract. 2018; 68(667):e81-e89.
19. Department of Health 2014 The Mandate: A mandate from the Government to NHS England: April 2014 – March 2015 https://www.gov.uk/government/uploads/system/uploads/attachment_data/file/383495/2902896_DoH_Mandate_Accessible_v0.2.pdf Accessed 4.7.18
20. Ridd MJ, King AJL, Le Roux E, et al. Systematic review of self-management interventions for people with eczema. Br J Dermatol. 2017; 177(3):719-734.
21. Hanlon P, Daines L, Campbell C. et al. Telehealth interventions to support self-management of long-term conditions: a systematic metareview of diabetes, heart failure, asthma, chronic obstructive pulmonary disease, and cancer. JMIR. 2017; 19:e172.

For peer review only - http://bmjopen.bmj.com/site/about/guidelines.xhtml

22. Sackett, DL, Rosenberg WM, Gray JM, et al. Evidence based medicine: what it is and what it isn't. BMJ. 1996; 312:71-2.
23. Greenhalgh T, Howick J, Maskrey N. "Evidence based medicine: a movement in crisis?" BMJ 348 (2014): g3725.
24. Brownson RC, Colditz GA, Proctor EK. eds. Dissemination and implementation research in health: translating science to practice. Oxford: Oxford University Press; 2017.
25. Vaucher C, Bovet E, Bengough T, et al. Meeting physicians' needs: a bottom-up approach for improving the implementation of medical knowledge into practice. Health Res Policy Syst. 2016; 14:49.
26. Ward V. 2017. Why, whose, what and how? A framework for knowledge mobilisers. Evid Policy. 2017; 13:477-497.
27. Ferlie E, Crilly T, Jashapara A, et al. Knowledge mobilisation in healthcare: a critical review of health sector and generic management literature. Soc Sci Med. 2012;74:1297-1304.
28. Gabbay J, Le May A. Practice-based evidence for healthcare: clinical mindlines. London: Routledge; 2010.
29. Plamondon K, Caxaj S. 2018. Toward Relational Practices for Enabling Knowledge-to-action in Health Systems: The Example of Deliberative Dialogue. Adv Nurs Sci. 2018; 41:18-29.
30. Gabbay J, le May A. Evidence based guidelines or collectively constructed "mindlines?" Ethnographic study of knowledge management in primary care. BMJ. 2004; 329:1013.
31. Polanyi M. The logic of tacit inference. Philos.1966; 41:1-18.
32. Nonaka I, Takeuchi H. The knowledge-creating company: How Japanese companies create the dynamics of innovation. Oxford: Oxford University Press; 1995.
33. Wieringa S, Greenhalgh T. 10 years of mindlines: a systematic review and commentary. Imp Sci. 2015; 10:45.
34. Hammersley M, Atkinson P. Ethnography: Principles in Practice. Abingdon: Routledge;2007.
35. Spradley JP. Participant observation. Illinois: Waveland Press; 2016.
36. Spradley JP. The ethnographic interview. Illinois: Waveland Press; 2016.
37. Cowdell F. That's how we do it we treat them all the same: An exploration of the experiences of patients, lay carers and health and social care staff of the care of people with dementia in acute hospital settings. Cambridge. Cambridge Publishing. 2013.
38. Tongco MD. Purposive sampling as a tool for informant selection. Ethnobotany Research and Applications. 2007. 31; 5:147-58.
39. O'Reilly M, Parker N. 'Unsatisfactory Saturation': a critical exploration of the notion of saturated sample sizes in qualitative research. Qual Res. 2013; 13:190-197.
40. Clinical Knowledge Summaries. Eczema – atopic https://cks.nice.org.uk/eczema-atopic Accessed 4.7.18
41. GP Notebook. Eczema http://www.gpnotebook.co.uk/simplepage.cfm?ID=-623902719 Accessed 4.7.18
42. Polkinghorne DE. Language and meaning: Data collection in qualitative research. J Couns Psychol, 2005; *52*(2):137.
43. Matua GA, Van Der Wal DM. Differentiating between descriptive and interpretive phenomenological research approaches. Nur Res. 2015; 22(6):22.
44. Finlay L. Phenomenology for psychotherapists: Researching the lived world. Oxford: Wiley Blackwell; 2011.
45. University of Hertfordshire 2017 Disadvantages of Ethnographic Research http://www.health.herts.ac.uk/immunology/Web%20programme%20-%20Researchhealthprofessionals/disadvantages_associated_with_et.htm. Accessed 4.7.18
46. Gabbay J, le May. Mindlines: making sense of evidence in practice. B J Gen Pract. 2016: 402-403.
47. Kahneman D. Thinking, Fast and Slow. London: Penguin; 2011.
48. Chandler CI, Jones C, Boniface G et al. Guidelines and mindlines: why do clinical staff over-dignose malaria in Tanzania? A qualitative study. Malar J 2008;7:53
49. Wieringa S, Engebretsen E, Heggen K, et al. How knowledge is constructed and exchanged in virtual communities of physicians: qualitative study of mindlines online. JMIR. 2018;20: e34.

For peer review only - http://bmjopen.bmj.com/site/about/guidelines.xhtml

50. Rosoff SM, Leone MC. (1989). The prestige of dermatologists. are they "last among equals"?Int J Dermatol. 1989; 28:377-380.
51. Rosoff SM, Leone MC (1991). The public prestige of medical specialties: Overviews and undercurrents. Soc Sci Med. 1991;32:321-326.
52. Creed PA, Searle J, Rogers ME. Medical specialty prestige and lifestyle preferences for medical students. Soc Sci Med. 2010;71:1084-8.
53. Cornwell J. Hard-earned lives: accounts of health and illness from East London:Routledge Kegan & Paul;1984.
54. Soriano LF, Sahota A, Jolliffe V. An evaluation of general practitioners learning preferences in dermatology. Education for Primary Care. 2017; 17:1-2.
55. Baron SE, Cohen SN, Archer CB. Guidance on the diagnosis and clinical management of atopic eczema. Clin Exp Dermatol. 2012; 37:Suppl 1, 7-12.
56. Le Roux, E, Powell K, Banks JP, et al. GPs' experiences of diagnosing and managing childhood eczema: a qualitative study in primary care. Br J Gen Pract. 2018. Feb 16:bjgp18X694529.
57. Lau R, Stevenson F, Ong BN, et al. Achieving change in primary care—causes of the evidence to practice gap: systematic reviews of reviews. Imp Sci. 2015;11:40.
58. National Institute for Health and Care Excellence (2018) NICE Quality and Outcomes Framework indicator https://www.nice.org.uk/standards-and-indicators/qofindicators Accessed 4.7.18
59. Roland M, Guthrie B. Quality and Outcomes Framework: what have we learnt? BMJ 2016;354:i4060
60. Reeve J, Dowrick CF, Freeman GK, et al. Examining the practice of generalist expertise: a qualitative study identifying constraints and solutions. JRSM short reports. 2013; 4(12):2042533313510155.
61. Bate L, Hutchinson A, Underhill J, et al. How clinical decisions are made. Br J Clin Pharmacol. 2012; 74:614-20.
62. Cunningham M, Shirley A. Mindlines in a digital age. Education for Primary Care. 2015; 26:293-6.
63. Levine D, Bleakley A. Maximising medicine through aphorisms. Med Educ. 2012; 46:153-162
64. McColl MA, Aiken A, Smith K, et al. Actionable nuggets: knowledge translation tool for the needs of patients with spinal cord injury. Can Fam Physician. 2015; 61:e240-e248.
65. Pharmaceutical Services Negotiating Committee Medicines Use Review http://psnc.org.uk/services-commissioning/advanced-services/murs/ Accessed 4.7.18
66. Pharmaceutical Services Negotiating Committee New Medicine Service http://psnc.org.uk/services-commissioning/advanced-services/nms/ Accessed 4.7.18
67. Pharmaceutical Services Negotiating Committee Minor Ailment Service http://psnc.org.uk/services-commissioning/locally-commissioned-services/en8-minor-ailments-service/ Accessed 4.7.18

For peer review only - http://bmjopen.bmj.com/site/about/guidelines.xhtml

Marked copy BMJ Open KM Practitioner mindlines R2 24.5.19

Table 1: Practitioner interview topic guide

Table 2: Demographic details of participants

Table 3: Complete dataset

Table 4: Example of data analysis process

Figure 1: Practitioner eczema mindlines

Sources of information underpinning practitioner eczema mindlines.

For peer review only

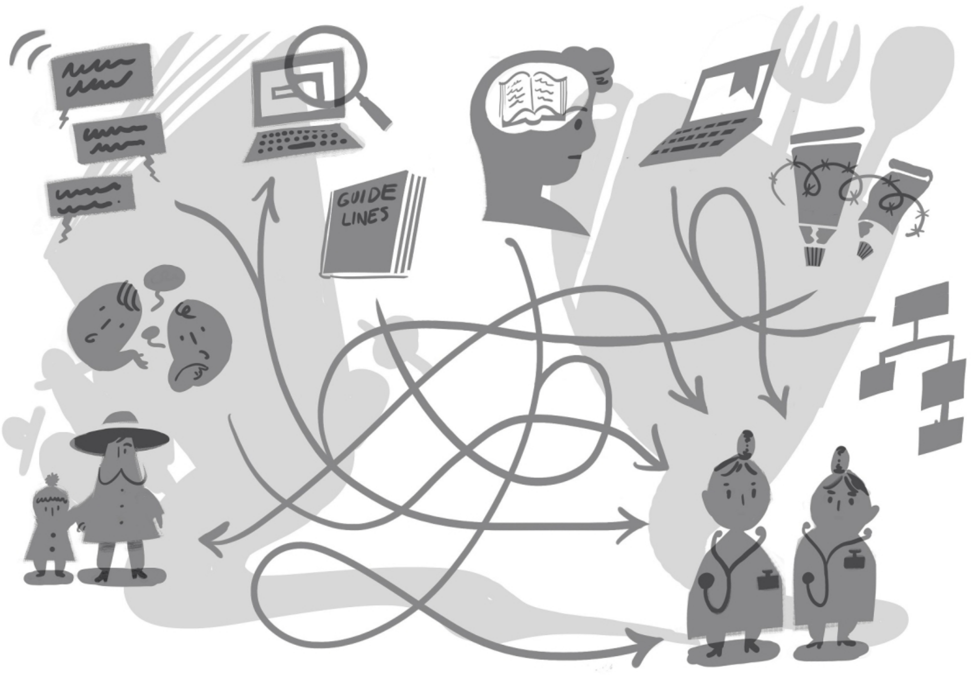

Practitioner eczema mindline

371x262mm (120 x 120 DPI)

BMJ Open Practitioner eczema mindlines COREQ 4.7.18

COREQ Statement

Knowledge mobilisation: An ethnographic study of the influence of practitioner mindlines on eczema self-management in primary care in the United Kingdom

Statement	Page no
**Domain 1: Research team and reflexivity**<br>*Personal Characteristics*<br>1. Interviewer/facilitator Which author/s conducted the interview or focus group?<br>I conducted all observation and interviews	6
2. Credentials What were the researcher's credentials? E.g. PhD, MD<br>DProf, RN	Title page
3. Occupation What was their occupation at the time of the study?<br>Professor of Nursing and Health Research	Title page
4. Gender Was the researcher male or female?<br>Female	Title page
5. Experience and training What experience or training did the researcher have?<br>I have extensive experience in qualitative research.	Title page
Relationship with participants<br>6. Relationship established Was a relationship established prior to study commencement?<br>I established rapport with each observational participant at each meeting and with interviewees at the time of interview.	6
7. Participant knowledge of the interviewer<br>What did the participants know about the researcher? e.g. personal goals, reasons for doing the research<br>I met the team prior to observation, provided an information sheet, explained about myself and the study and answered questions. I gave interview participants an information sheet prior to interviews and answered any questions, I reiterated this information at the beginning of each interview.	6
8. Interviewer characteristics What characteristics were reported about the interviewer/facilitator? e.g. Bias, assumptions, reasons and interests in the research topic<br>Participants were aware, and it is reported in the manuscript, that I am a Registered Nurse with an interest in how eczema knowledge is developed and shared between patients and practitioners in primary care and that this was a publically funded study.	8
**Domain 2: study design**<br>*Theoretical framework*<br>9. Methodological orientation and theory<br>What methodological orientation was stated to underpin the study? e.g. grounded theory, discourse analysis, ethnography, phenomenology, content analysis<br>In the methods section I explain that this is an ethnographic study using observation and interviews and that data analysis followed an ethnographic approach through the lenses of mindlines and self-management.	5
Participant selection<br>10. Sampling How were participants selected? e.g. purposive, convenience, consecutive, snowball<br>I collected observational data during 250 hours in the practice. I selected interview participants using maximum variation purposive sampling to ensure a mix of profession, gender and years in practice.	6
11. Method of approach How were participants approached? e.g. face-to-face, telephone, mail, email<br>Observation participants were recruited at the beginning of each encounter with the practice. I recruited interview participants from the practice and local pharmacy.	6
12. Sample size How many participants were in the study?<br>Observation involved many participants and 16 interviews were completed	6
13. Non-participation How many people refused to participate or dropped out? Reasons?<br>On occasion I exited consultations at the request of the patient, practitioner or of my own volition<br>A few practitioners declined to take part in interviews due to time constraints.	5
Setting<br>14. Setting of data collection Where was the data collected? e.g. home, clinic, workplace<br>Observational data was collected in a GP practice. Interviews were conducted in their workplace.	6
15. Presence of non-participants Was anyone else present besides the participants and researchers?	6

BMJ Open Practitioner eczema mindlines COREQ 4.7.18

Observational data involved numerous individuals. Interviews were conducted individually.	
16. Description of sample What are the important characteristics of the sample? e.g. demographic data, date<br>Interview participants were sampled by profession, gender and years in practice.	Table 2
Data collection<br>17. Interview guide Were questions, prompts, guides provided by the authors? Was it pilot tested?<br>A topic guide was used for interviews	Table 1
18. Repeat interviews Were repeat interviews carried out? If yes, how many?<br>No repeat interviews were carried out	NA
19. Audio/visual recording Did the research use audio or visual recording to collect the data?<br>Interviews were audio recorded and observational data recorded in field notes	6
20. Field notes Were field notes made during and/or after the interview or focus group?	6
21. Duration What was the duration of the interviews or focus group?<br>Interviews lasted from 22-40 minutes	6
22. Data saturation Was data saturation discussed?<br>Data sufficiency was achieved when no new sources of knowledge were identified in interviews.	8
23. Transcripts returned Were transcripts returned to participants for comment and/or correction?<br>No	NA
**Domain 3: analysis and findings**<br>*Data analysis*<br>24. Number of data coders How many data coders coded the data?<br>I analysed the data independently and subsequently discussed with research, clinical and lay colleagues who corroborated initial interpretations.	8
25. Description of the coding tree Did authors provide a description of the coding tree?<br>No	NA
26. Derivation of themes Were themes identified in advance or derived from the data?<br>Themes were derived inductively from the data	8
27. Software What software, if applicable, was used to manage the data?<br>No	NA
28. Participant checking Did participants provide feedback on the findings?<br>No	NA
Reporting<br>29. Quotations presented Were participant quotations presented to illustrate the themes / findings? Was each quotation identified? e.g. participant number<br>Quotations are provided and profession of participant is identified.	9 - 14
30. Data and findings consistent Was there consistency between the data presented and the findings?<br>Yes.	9 - 14
31. Clarity of major themes Were major themes clearly presented in the findings?<br>Yes.	9 - 14
32. Clarity of minor themes Is there a description of diverse cases or discussion of minor themes?<br>Yes, I present a spectrum of practitioners and variations of mindlines.	9 - 14

For peer review only
